# Genetic and epigenetic signatures for improved breeding of cultivated blueberry

**DOI:** 10.1093/hr/uhae138

**Published:** 2024-05-14

**Authors:** Zejia Wang, Wanchen Zhang, Yangyan Zhou, Qiyan Zhang, Krishnanand P Kulkarni, Kalpalatha Melmaiee, Youwen Tian, Mei Dong, Zhaoxu Gao, Yanning Su, Hong Yu, Guohui Xu, Yadong Li, Hang He, Qikun Liu, Haiyue Sun

**Affiliations:** State Key Laboratory of Protein and Plant Gene Research, School of Advanced Agricultural Sciences, Peking University, No.5 Yiheyuan Road, Haidian District, Beijing 100871, China; Jilin Provincial Laboratory of Crop Germplasm Resources, College of Horticulture, Jilin Agricultural University, No. 2888 Xincheng Street, Economic Development District, Changchun 130118, China; State Key Laboratory of Protein and Plant Gene Research, School of Advanced Agricultural Sciences, Peking University, No.5 Yiheyuan Road, Haidian District, Beijing 100871, China; State Key Laboratory of Protein and Plant Gene Research, School of Advanced Agricultural Sciences, Peking University, No.5 Yiheyuan Road, Haidian District, Beijing 100871, China; Department of Agriculture and Natural Resources, Delaware State University, Dover, DE 19901, USA; Department of Agriculture and Natural Resources, Delaware State University, Dover, DE 19901, USA; Jilin Provincial Laboratory of Crop Germplasm Resources, College of Horticulture, Jilin Agricultural University, No. 2888 Xincheng Street, Economic Development District, Changchun 130118, China; Jilin Provincial Laboratory of Crop Germplasm Resources, College of Horticulture, Jilin Agricultural University, No. 2888 Xincheng Street, Economic Development District, Changchun 130118, China; State Key Laboratory of Protein and Plant Gene Research, School of Advanced Agricultural Sciences, Peking University, No.5 Yiheyuan Road, Haidian District, Beijing 100871, China; State Key Laboratory of Protein and Plant Gene Research, School of Advanced Agricultural Sciences, Peking University, No.5 Yiheyuan Road, Haidian District, Beijing 100871, China; Institute of Botany, Jiangsu Province and Chinese Academy of Sciences, Nanjing 210014, China; College of Life and Health, Dalian University, Dalian 116622, China; Jilin Provincial Laboratory of Crop Germplasm Resources, College of Horticulture, Jilin Agricultural University, No. 2888 Xincheng Street, Economic Development District, Changchun 130118, China; State Key Laboratory of Protein and Plant Gene Research, School of Advanced Agricultural Sciences, Peking University, No.5 Yiheyuan Road, Haidian District, Beijing 100871, China; State Key Laboratory of Protein and Plant Gene Research, School of Advanced Agricultural Sciences, Peking University, No.5 Yiheyuan Road, Haidian District, Beijing 100871, China; Jilin Provincial Laboratory of Crop Germplasm Resources, College of Horticulture, Jilin Agricultural University, No. 2888 Xincheng Street, Economic Development District, Changchun 130118, China

## Abstract

Blueberry belongs to the *Vaccinium* genus and is a highly popular fruit crop with significant economic importance. It was not until the early twentieth century that they began to be domesticated through extensive interspecific hybridization. Here, we collected 220 Vaccinium accessions from various geographical locations, including 154 from the United States, 14 from China, eight from Australia, and 29 from Europe and other countries, comprising 164 *Vaccinium corymbosum*, 15 *Vaccinium ashei*, 10 lowbush blueberries, seven half-high blueberries, and others. We present the whole-genome variation map of 220 accessions and reconstructed the hundred-year molecular history of interspecific hybridization of blueberry. We focused on the two major blueberry subgroups, the northern highbush blueberry (NHB) and southern highbush blueberry (SHB) and identified candidate genes that contribute to their distinct traits in climate adaptability and fruit quality. Our analysis unveiled the role of gene introgression from *Vaccinium darrowii* and *V. ashei* into SHB in driving the differentiation between SHB and NHB, potentially facilitating SHB’s adaptation to subtropical environments. Assisted by genome-wide association studies, our analysis suggested *VcTBL44* as a pivotal gene regulator governing fruit firmness in SHB. Additionally, we conducted whole-genome bisulfite sequencing on nine NHB and 12 SHB cultivars, and characterized regions that are differentially methylated between the two subgroups. In particular, we discovered that the β-alanine metabolic pathway genes were enriched for DNA methylation changes. Our study provides high-quality genetic and epigenetic variation maps for blueberry, which offer valuable insights and resources for future blueberry breeding.

## Introduction

Blueberries refer to blue-fruited plants in the *Vaccinium* genus of the Ericaceae family. Most cultivated blueberries belong to the section *Cyanococcus* [1] (Fig. S1, see online supplementary material). Blueberries are highly nutritious and provide numerous benefits for human health [2, 3]. The global blueberry industry has experienced steady growth over the past decade, driven by increasing consumer demand for healthy and nutritious foods. According to data from TRIDGE (https://www.tridge.com/intelligences/billberry/production), the global total blueberry production was approximately 823 300 tons in 2019, which is 2.5 times the amount in 2010.

Blueberries are native to North America and have been a part of the diet of indigenous peoples for thousands of years. However, cultivation of blueberries did not begin until the early twentieth century [4]. Modern cultivated blueberries can be categorized into three groups: highbush blueberry (HB), lowbush blueberry (LB), and rabbiteye blueberry (RB). Highbush blueberries can be further divided into northern highbush (NHB), southern highbush (SHB), and half-high blueberry (HHB); these subgroups differ by their interspecific hybridization history [5]. Compared to most other cultivated crops and fruits, blueberries have a much shorter breeding and cultivation history of just over 100 years [6], which involved extensive interspecific hybridization [7]. For example, the interspecific hybridization among *V. corymbosum*, *V. darrowii*, and *V. ashei* led to development of the SHB subgroup that displays significantly enhanced adaptation to warmer climates in low-latitude zones [5]. The rapid expansion of blueberry cultivation across a wide range of latitudes through interspecific hybridization has made blueberry an ideal research model for studying the molecular mechanisms associated with *de novo* domestication of crops.

In recent years, significant advancements in sequencing technology have enabled researchers to dissect population structures and uncover genetic variation underlying important agronomic traits [8–11]. Reference genome sequences for both tetraploid and diploid blueberries are available [12–14]. Utilizing these high-quality reference genomes, researchers have employed reduced-representation genome sequencing at the population level to unravel gene flow events in cultivated blueberries and extensively identify selective genomic regions [5, 15, 16]. Recently, the pan-genomes of cranberry and highbush blueberry have been successfully constructed, providing valuable resources for future genetic research and breeding efforts [17]. In addition to genetic variation, epigenetic modifications are increasingly recognized as important factors in crop domestication and improvement. Recent studies revealed the impact of DNA methylation in regulating important agronomic traits, such as carbohydrate metabolism in soybean [18], fruit development in tomato [19], drought resistance in maize [20], reproductive development in rice [21], and flowering transition in cotton, etc. [22]. Thus, a thorough understanding of the genetic and epigenetic divergence among different blueberry cultivars is essential for a complete understanding of the significant progress in blueberry improvement made through interspecific hybridization and is also critical for future efforts to perform targeted breeding and trait improvement in blueberry.

In this study, we performed whole-genome re-sequencing of 220 accessions from the *Vaccinium* genus, covering all major blueberry cultivated subgroups and whole-genome bisulfite sequencing of nine NHB and 12 SHB cultivars. Through analysis of genetic and epigenetic variation, we reconstructed the breeding history of cultivated blueberries. We elucidated the genetic basis of subtropical adaptation of the SHB subgroup facilitated by gene introgression from *V. darrowii* and *V. ashei*. Furthermore, through a genome-wide association study (GWAS) we found that differentiation of the *VcTBL44*, a gene of cell wall modifying function in the SHB subgroup, likely contributed to an increase in fruit firmness. Through population-level DNA methylation analysis, we discovered that differential methylation in the SHB subgroup compared to the NHB subgroup significantly affected the β-alanine metabolism pathway, potentially facilitating SHB’s adaptation to warmer environments. Together, our findings shed light on the genetic and epigenetic signatures associated with blueberry improvement through interspecific hybridization and provide valuable resources for the genetic improvement of this economically important fruit.

## Results

### Genome variation map of blueberry

We obtained 220 accessions belonging to the *Vaccinium* genus from major blueberry-producing regions across the globe (Fig. S2A, see online supplementary material). Among these, 24 accessions belonged to species closely related to modern cultivated blueberries, comprising 12 accessions of *V. macrocarpon* (Cranberry, CB), one of *V. darrowii*, two of *V. vitis-idaea*, two of *V. uliginosum*, one of *V. reticulatum*, one of *V. koreanum*, one of *V. oldhami*, one of *V. bracteatum*, one of *V. myrtillus L.*, one of *V. pallidum*, and one of *V. carlesii*. The remaining 196 accessions were blueberry cultivars, specifically, 81 NHBs, 83 SHBs, seven HHBs, 15 RBs, and 10 LBs, based on their released records. (Fig. S2B, Table S1, see online supplementary material). Together, these cultivars represent germplasms from all major subgroups of cultivated blueberries.

We obtained approximately 5.57 Tb of whole-genome re-sequencing data for the 220 *Vaccinium* accessions (Table S1, see online supplementary material). Considering that 164 out of 220 (75%) accessions were either NHB or SHB cultivars, with 161 of them confirmed as tetraploids based on their release records and 159 predicted as tetraploids by nQuire analysis (Table S1, see online supplementary material) [23], we chose the tetraploid NHB cultivar ‘Draper’ as our designated reference genome [12]. Following the convention of previous studies, the 12 longest scaffolds among homologous chromosomes from ‘Draper’ were chosen as reference genome [5, 12, 16] (see Discussion). These re-sequencing reads covered 93.22% of the reference genome with an average depth of 35-fold (Table S1, see online supplementary material). After stringent filtering (see Materials and methods), we retained a total of 3 246 135 high-quality SNPs for further analysis, with an average of 6.62 SNPs per kb (Table S2, see online supplementary material). Among these SNPs, 341 567 were predicted to have significant impacts on the gene products, including 295 993 variants resulting in missense variations, 4741 causing start and stop codon changes, and 40 833 affecting splicing sites (Table S2, see online supplementary material). The high-density genomic variation map that we obtained will be a valuable resource for blueberry breeding and genetic improvement (see Data availability).

### Population structure

**Figure 1:**
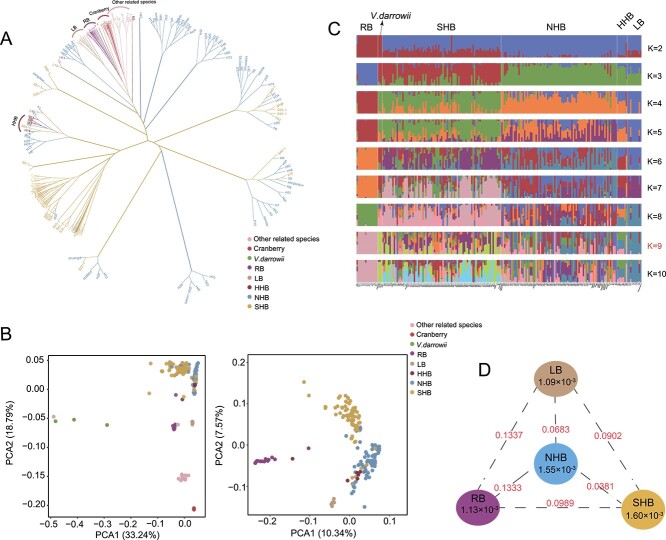
Population structure and genetic diversity of blueberry accessions. **A** Maximum likelihood phylogenetic tree of 222 blueberry accessions. HHB: half-high blueberry; LB: lowbush blueberry; NHB: northern highbush blueberry; RB: rabbiteye blueberry; SHB: southern highbush blueberry. **B** Principal component analysis of the first two components (PC1 and PC2) for all accessions and major cultivated blueberry accessions. PC1, first principal component; PC2, second principal component. **C** Population structure analysis of cultivated blueberry accessions and *V. darrowii* given different cluster numbers (K = 2–10). The y axis quantifies subgroup membership, and the x axis shows the different accessions. The K value marked in red is the optimal K value determined based on the CV error. **D** Nucleotide diversity (π) and population divergence (*F*_ST_ value) across four cultivated blueberry subgroups (calculated from the diploid model). The values in the circles represent the nucleotide diversity (π) of the groups (brown, orange, blue, and purple circles represent the LB, SHB, NHB, and RB subgroups, respectively), and the value between each pair indicates population divergence (*F*_ST_ value).

We performed a population structure analysis using 222 accessions, including the 220 sequenced in this study and two published accessions of *V. darrowii* [13, 14]. To investigate the genetic and evolutionary relationship between cultivated blueberries and other closely related species in the *Vaccinium* genus, a maximum likelihood (ML) tree was constructed using 609 456 high-quality SNPs in linkage disequilibrium (LD) (Fig. 1A). The analysis indicated that most cultivated blueberry subgroups were clearly distinguished from cranberries and other related species (Fig. 1A). The RB was closest to the related species of cultivated blueberries. Similar to findings from previous studies [5, 15, 24], NHB and SHB did not exhibit distinct phylogenetic separation. Many cultivars identified as NHB appeared in clusters of SHB cultivars, and vice versa. In the breeding process, the HHB cultivar was primarily a hybrid between NHB and LB [1]. Consequently, HHB samples were positioned close to both NHB and LB on the phylogenetic tree (Fig. 1A).

Principal component analysis (PCA) of the 222 accessions indicated a distinct separation between cultivated blueberries and other closely related species, including cranberries and *V. darrowii* (Fig. 1B). To further explore the relationship among the cultivated blueberry subgroups, a second round of PCA was conducted on the *Vaccinium* population after removing closely related cultivated blueberry species (Fig. 1B). The results supported our findings from the phylogenetic tree analysis, indicating a closer genetic relationship between the NHB and SHB subgroups compared to other blueberry subgroups. Notably, the HHB subgroup exhibited a scattered distribution between the NHB and LB accessions (Fig. 1B), supporting the notion that the HHB cultivar was primarily generated through hybridization between the NHB and LB cultivars during the breeding process.

These phylogenetic relationships were further supported by model-based analyses of population admixture, which revealed that LB and RB cultivars each form a distinct, independent clade (Fig. 1C). Notably, LB, known for its increased resistance to cold and drought stress compared to other subgroups, has been utilized as breeding material for blueberry improvement [25]. In line with this notion, our population admixture analysis also indicated the presence of genetic infiltration of LB in HHB (Fig. 1C). The two largest cultivated subgroups, NHB and SHB, exhibited more complex patterns of genetic admixture. RB (*V. ashei*) and *V. darrowii* were also breeding parents of SHB [1]; thus, the lineage of both of these subgroups could be traced within the SHB lineage (Fig. 1C). Notably, based on the cross-validation (CV) errors (Fig. S3A, see online supplementary material), the optimal value of K was 9. This result aligned with previous study suggesting that modern cultivated blueberries have *V. darrowii*, *V. corymbosum*, *V. angustifolium*, and *V. virgatum* as the genomic backbone, while incorporating the genomic characteristics of *V. constablaei, V. elliottii, V. myrtilloides, V. pallidum*, and *V. tenellum* [5, 26]*.*

Next, we analysed and compared nucleotide diversity and genetic differentiation among different blueberry subgroups. We found that the nucleotide diversity of NHB and SHB was slightly higher than that of LB and RB (Fig. 1D). As the sample size of NHB and SHB is larger than that of RB and LB, we repeated the analysis with the sample size of NHB and SHB adjusted to be comparable with that of RB and LB, and obtained the same results (Fig. S3B, see online supplementary material). Due to the complex ploidy of blueberries, nucleotide diversity calculated for species with different ploidies may exhibit biases [27, 28]. Therefore, we focused our analysis on the NHB and SHB subgroups, both of which are dominated by cultivars with tetraploid genomes (Table S1, see online supplementary material). Consistent with previous discovery using reduced-representation genome sequencing [5, 16], we found that NHB and SHB exhibited negligible genetic differentiation (*F*_ST_=0.0381, Fig. 1D). Nevertheless, we discovered that the nucleotide diversity of SHB was slightly greater than that of NHB (Fig. 1D). Given the historical cultivation record showing that the breeding of SHB for improved subtropical adaptation has involved interspecific hybridization among *V. corymbosum*, *V. darrowii,* and *V. ashei* [13, 14], we speculated that the higher genetic diversity of SHB may be attributed to introgression from these other *Vaccinium* species during the breeding process.

### Gene introgression facilitated population differentiation and subtropical adaptation of the SHB subgroup

To dissect the causes of the higher nucleotide diversity observed in SHB, we first characterized genetic differences between NHB and SHB on a genome-wide scale. Overall, the population differentiation between NHB and SHB was not pronounced (*F*_ST_ = 0.0381, Fig. 1D). Nonetheless, specific regions across the genome clearly demonstrated population differentiation (Fig. S4A, see online supplementary material). The genomic distribution of population differentiation between NHB and SHB appeared to be uneven. By associating these regions with major genomic elements, we discovered a potential positive correlation between the greatest sequence diversity (π) of NHB and SHB and the density of LTR-retrotransposons (LTR-RTs) (Fig. S4A and B, see online supplementary material). Genome regions with high densities of transposons and repeats are known to negatively affect mapping quality of short sequencing reads. Therefore, the difference in sequence diversity observed between NHB and SHB may be attributed to differences in the mapping ratio and coverage for these regions. Further examination revealed no significant differences in the alignment depth and coverage between NHB and SHB in these transposon-rich regions (Fig. S4C, see online supplementary material). However, for both NHB and SHB, the mapping ratios in these regions were generally lower than those for other genomic regions (Fig. S4C, see online supplementary material); therefore, the impact of mapping bias cannot be dismissed entirely.

**Figure 2:**
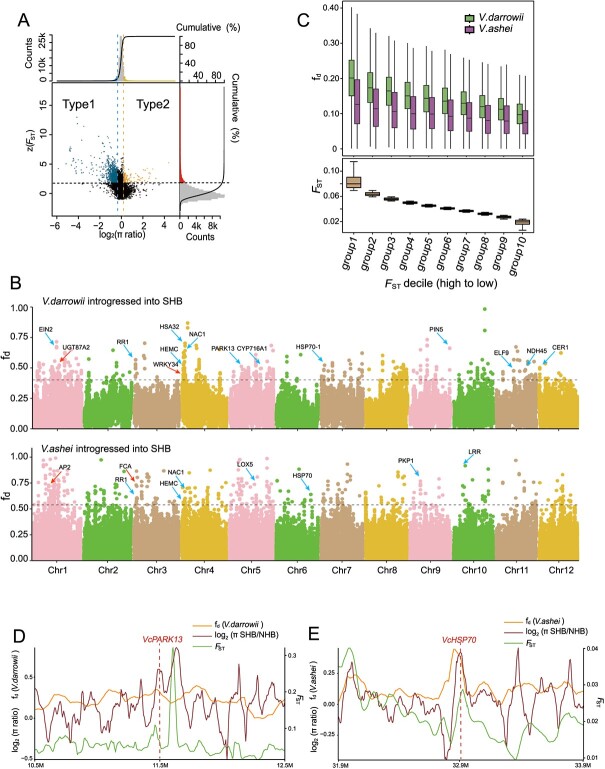
Identification of introgression signals from *V. darrowii* and *V. ashei* into the SHB subgroup. **A** Distribution of π ratios (πNHB/πSHB) and population differentiation index (*F*_ST_ values). Points located to the left of the blue vertical dashed line indicate regions of the bottom 5% of the π ratios (log_2_ (π ratio) < −0.37). Points located to the right of the orange vertical dashed line indicate regions of the top 5% of the π ratios [log_2_ (π ratio) >0.21]. Points above the horizontal dashed line indicate regions of the top 5% of *F*_ST_ values [z (*F*_ST_ value) > 1.79]. The count distribution and cumulative value curves are displayed on both the top and right sides of the figure. **B** Genome-wide distribution of f_d_ values calculated for 50-kb sliding windows with a 5-kb step across the genomes. The upper graph represents the introgression from *V. darrowii* into the SHB subgroup, while the lower graph represents the introgression from *V. ashei* into the SHB subgroup. The black dashed line represents the top 1% of values. The red arrows indicate candidate genes related to flowering. The blue arrows indicate candidate genes related to environmental adaptation and stress resistance. **C** Boxplots showing f_d_ values (upper panel) and *F*_ST_ values (lower panel) in different genomic regions. The blueberry genomic regions were divided into ten deciles based on their *F*_ST_ values, arranged from high to low. **D** The distribution of π ratios (πSHB/πNHB, brown curve), *F*_ST_ values (green curve) and f_d_ values (from *V. darrowii* to the SHB subgroup, orange curve) in the vicinity of the *VcPARK13* on chromosome 5, with *VcDEG14* represented by the red dashed line. **E** Distribution of π ratios (πSHB/πNHB, brown curve), *F*_ST_ values (green curve), and f_d_ values (from *V. ashei* to SHB subgroup, orange curve) in the vicinity of *VcHSP70* on chromosome 6, with the *VcHSP70* represented by the red dashed line.

Next, we tested whether SHB exhibits overall higher nucleotide diversity in regions of greater sequence diversity between SHB and NHB. To answer this question, we identified intervals in SHB with clear differentiation and a high SHB-to-NHB nucleotide diversity ratio (Type 1 interval, top 5% of *F*_ST_ value and top 5% of the π_SHB_/π_NHB_), as well as intervals in SHB with clear differentiation and a low SHB-to-NHB nucleotide diversity ratio (Type 2 interval, top 5% of *F*_ST_ value and bottom 5% of the π_SHB_/π_NHB_, Fig. 2A). Interestingly, we found that the number of Type 1 intervals was much greater than that of Type 2 intervals (23.06 Mb vs. 4.84 Mb, Fig. 2A). We speculated that the larger number of Type 1 intervals in SHB resulted from gene introgression through interspecies hybridization. To test this idea, we utilized the f_d_ value to analyse gene introgression from *V. darrowii* and *V. ashei* into SHB (see Materials and methods) [29]. Our observations indicated substantial gene introgression from both *V. darrowii* and *V. ashei* into SHB (Fig. 2B; Table S3, see online supplementary material). We further divided the whole genome into 10 equally sized segments based on varying levels of population differentiation. Subsequently, we quantified gene introgression from *V. darrowii* and *V. ashei* into SHB within each segment. As expected, we found that regions that displayed greater population divergence between SHB and NHB also showed increased gene introgression from both *V. darrowii* and *V. ashei* (Fig. 2C). This finding emphasized the contribution of gene introgression to enhancing genetic diversity in SHB.

Introgression of *V. darrowii* and *V. ashei* is believed to have resulted in changes in plant chilling requirement, flowering time, as well as heat and drought tolerance in SHB, improving its adaptability to warmer subtropical climates [1, 13, 14]. In the high introgression regions (top 1%) of *V. darrowii* and *V. ashei*, we identified the flowering-related genes *VcWRKY34*, *VcAP2*, and *VcFCA,* as well as the heat resistance-related genes *VcHSP70*, *VcPARK13*, and *VcLOX5* (Fig. 2B). Previous studies have indicated that *PARK13* is induced by heat stress and is involved in the degradation of misfolded proteins [30]. We observed that gene introgression from *V. darrowii* significantly increased nucleotide diversity in regions near *VcPARK13* (Fig. 2D). In addition, *HSP70*, functions as a molecular chaperone to assist in protein folding and assembly, and it plays a crucial role in plant heat defense [31]. Similarly, genetic introgression from *V. ashei* was strongly enriched at the *VcHSP70* locus and was accompanied by increased nucleotide diversity in SHB relative to NHB (Fig. 2E).

Both *V. darrowii* and *V. ashei* have strong subtropical adaptability. We further examined the genes present in strong introgression regions (top 1%) from either *V. darrowii* and *V. ashei* and observed a significant overlap in these genes between the two varieties (Fig. S5A, see online supplementary material). This is likely due to the fact that these genes are associated with alleles that are commonly present in both the *V. darrowii* and *V. ashei* genome, and therefore, the exact source of genetic introgression can be either one of them. Gene Ontology (GO) enrichment analysis of the overlapping set of 121 genes revealed their primary association with saponin and brassinosteroid metabolism, as well as auxin response (Fig. S5B, see online supplementary material). Previous studies have shown that brassinosteroids play important roles in regulating plant responses to drought and heat stresses, as well as nutrient deficiency [32, 33]. To summarize, our analysis indicates that gene introgression from *V. darrowii* and *V. ashei* into SHB has contributed to increased population differentiation between NHB and SHB, facilitating SHB’s adaptation to subtropical conditions.

In a previous blueberry population study, the genetic background of 81 accessions of *V. darrowii* was characterized by genotyping-by-sequencing (GBS) [15]. We also examined the SNP profile generated for these 81 *V. darrowii* accessions and compared the f_d_ values calculated in regions detected in both our and their studies. We observed an overall positive correlation between f_d_ calculated using our SNP datasets (Fig. S6, x-axis, see online supplementary material) and theirs (Fig. S6, y-axis, see online supplementary material). This analysis further supports the view that there was genetic introgression from *V. darrowii* into SHB during SHB breeding.

### *VcTBL44* is associated with increased fruit firmness in the SHB subgroup

**Figure 3:**
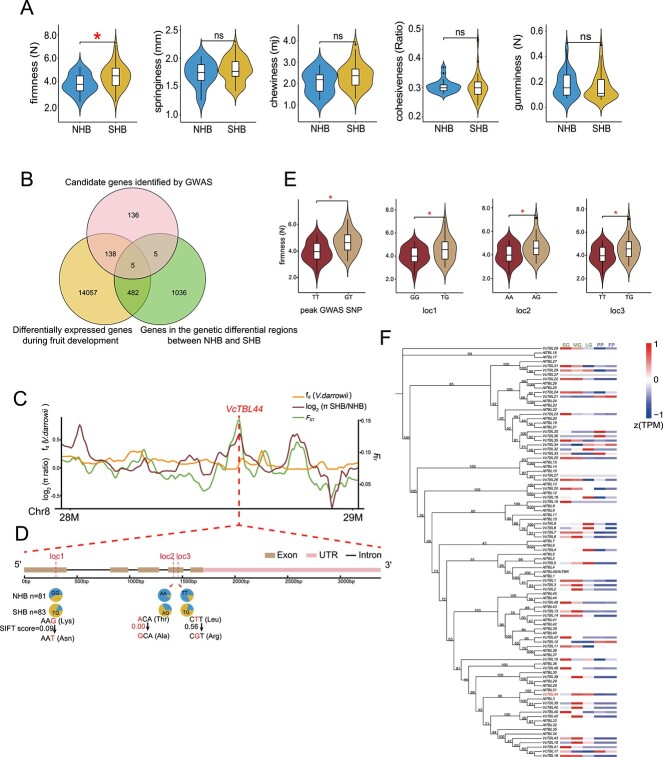
Identification of key genes affecting fruit firmness. **A** Comparisons of firmness, springiness, chewiness, cohesiveness, and gumminess (from left to right) between the NHB and SHB subgroups. Statistically significant differences between the groups were assessed using a Mann–Whitney–Wilcoxon test (two-sided), with significant results (*P*-value <0.05) indicated by a red asterisk. ‘ns’ indicates no significant difference. **B** Venn diagram of candidate genes identified by GWAS analysis and genes differentially expressed during fruit development and genes in the genetic differential regions between NHB and SHB. **C** The distribution of π ratios (πSHB/πNHB, brown curve), f_d_ values (orange curve), and *F*_ST_ values (green curve) in the vicinity of *VcTBL44* on chromosome 8, with the position of *VcTBL44* represented by the red dashed line. **D** Missense variations in *VcTBL44* and their allelic frequency in the NHB and SHB subgroups. Red dashed lines indicate the positions of missense variants. The pie chart at each position represents allelic frequency within the NHB (upper) and SHB (lower) subgroups. Below each pie chart, variations at the codon level are displayed and highlighted in red to indicate the specific nucleotide change. The SIFT score was utilized to assess the impact of the variant on protein function, with smaller values indicating a greater impact. The value <0.05 is considered to have a significant effect. **E** Fruit firmness of accessions carrying different alleles of significant peak SNP and missense variants in *VcTBL44*. Location code matches with those in (**D**). Statistically significant differences between the groups were assessed using a Mann–Whitney–Wilcoxon test (two sided), with significant results (*P*-value<0.05) indicated by a red asterisk. **F** Phylogenetic tree displaying the relationship between blueberry and *Arabidopsis thaliana TBL* homologs, with bootstrap values indicated on the branches. The heatmap illustrates the expression levels of *TBLs* in blueberries during fruit development. SG indicates small green; MG indicates medium green; LG indicates large green; PP indicates partial purple; and FP indicates full purple. Genes without expression are depicted as grey bands. *VcTBL44* is highlighted in red.

Fruit quality is one of the most important traits in blueberry breeding [6]. We investigated whether any distinct changes in fruit quality occurred during the differentiation between NHB and SHB. We selected 50 cultivars (including 18 NHB and 32 SHB) based on their genetic relationships to represent various evolutionary branches of NHB and SHB, considering the proportional distribution of cultivars within each branch (Fig. S7, see online supplementary material). We measured their fruit firmness, springiness, chewiness, cohesiveness, and gumminess (Fig. 3A; Table S4, see online supplementary material). Our findings suggested that the fruit characteristics of NHB and SHB were largely similar, except that SHB exhibited higher firmness compared to NHB (Fig. 3A). Similar to our findings, a previous study also reported higher fruit firmness in SHB and RB compared to NHB [34].

High fruit firmness is important for maintaining fruit quality during long-distance shipping, making it a desirable trait for blueberry breeding [34]. To unravel the genes influencing blueberry fruit firmness, we conducted a transcriptome analysis for fruits at different developmental stages, including small green (SG, 15 days after flowering), middle green (MG, 30 days after flowering), large green (LG, 45 days after flowering), partial purple (PP, 60 days after flowering), and full purple (FP, 75 days after flowering) (Fig. S8, see online supplementary material). The differentially expressed genes (DEGs) were defined as those showing differential expression in pair-wise comparison between any two stages. As a result, 14 682 DEGs were identified (Table S5, see online supplementary material). Gene Ontology analysis suggests that these genes are associated with biological membranes, ribosomes, chloroplast plastid thylakoid, and carbohydrate metabolism (Fig. S9, see online supplementary material). To further identify genes related to the genetic differentiation between NHB and SHB, we examined the genomic location of these developmental DEGs focused on regions showing distinct genetic differentiation between NHB and SHB (Type 1 interval and Type 2 interval). The analysis results in a total of 487 genes (Table S5, see online supplementary material). Gene Ontology analysis revealed no enrichments for specific gene annotations, suggesting that a diverse category of genes regulating fruit development were introgressed during SHB improvements.

Next, we conducted GWAS and identified 284 candidate genes showing significant association with higher fruit firmness (Fig. S10, Table S5, see online supplementary material). Interestingly, cross-comparison using the GWAS candidates further narrowed down the initial 487 candidate genes to five members, including *USUALLY MULTIPLE ACIDS MOVE IN AND OUT TRANSPORTERS 41 (VcUMAMIT41)*, *CYTOCHROME P450, FAMILY 94, SUBFAMILY C, POLYPEPTIDE 1 (VcCYP94C1)*, *GLYCOSYLPHOSPHATIDYLINOSITOL-ANCHORED LIPID PROTEIN TRANSFER 32 (VcLTPG32)*, *TRICHOME BIREFRINGENCE-LIKE 44 (VcTBL44)*, and *snap_masked-VaccDscaff22-processed-gene-241.11* (Fig. 3B).

Among the five candidate genes identified in the above analysis, *VcTBL44* caught our attention. Members of the *TBL* gene family encode O-acetyltransferases, which are involved in modifying cell wall polysaccharides, such as pectin [35]. Studies in tomatoes have demonstrated the role of *TBL* in promoting fruit softening [35, 36]. The SNP we identified as being associated with blueberry fruit firmness was located 7.3 kb downstream of *VcTBL44*, within the same LD block (Fig. S11, see online supplementary material). *VcTBL44* was observed to have significant population differentiation, with increased genetic diversity within the SHB subgroup (Fig. 3C). In addition to the peak GWAS SNP, comparison of the coding sequences of *VcTBL44* between NHB and SHB cultivars revealed three missense variants (loc1, 2, and 3), all of which showed differing allele distribution frequencies in NHB and SHB subgroups (Fig. 3D). While NHB cultivars tend to be homozygous for each of the three missense variants, SHB cultivars are predominantly heterozygous at these loci (Fig. 3D). To determine the impact of these missense variants on protein function, we assessed their sequence homology as well as the physical properties of the encoded amino acids using the SIFT algorithm (see Materials and methods) [37]. The SIFT analysis predicted that the missense variation at loc2 strongly impacts *VcTBL44* protein functionality, which may underlie the increased fruit firmness in SHB (Fig. 3D). Furthermore, cultivars that are heterozygous at these loci show increased fruit firmness compared to cultivars carrying homozygous alleles (Fig. 3E). Because NHB and SHB cultivars are primarily tetraploids (Table S1, see online supplementary material), we analysed the allele distribution frequency at each of these loci for all NHB and SHB cultivars. Interestingly, the allele frequencies at loc2 and loc3 appear to correlate with each other in each of the SHB cultivars, but not with that at loc1 in a given cultivar (Fig. S12, see online supplementary material).

In addition to *VcTBL44*, we also investigated the expression of other members of the *VcTBL* family (Table S6, see online supplementary material). We found that expression of *VcTBLs*, including *VcTBL44*, was primarily detected in the early stages during fruit development and gradually decreased as the fruits ripened (Fig. 3F). These results suggested that *VcTBL44*, and perhaps also other *VcTBL*s, play an important role in regulating blueberry fruit texture during fruit development.

### DNA methylation pattern differs between the NHB and SHB subgroups

**Figure 4:**
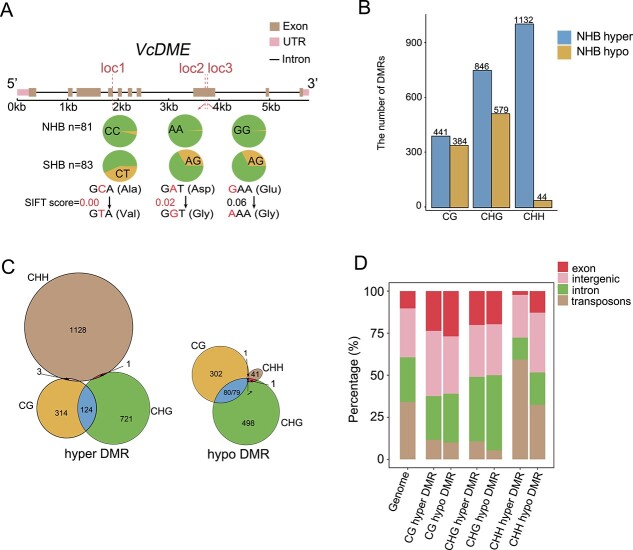
The impacts of differential selection between NHB and SHB on DNA methylation. **A** Missense variants in *VcDME* and their allele frequency in two blueberry subgroups. The red dashed lines indicate the positions of missense variants. The pie chart at each position represents allele frequency within the NHB (upper) and SHB (lower) subgroups. Below each pie chart, variations at the codon level are displayed and highlighted in red to indicate the specific nucleotide change. The SIFT score was utilized to assess the impact of the variant on protein function, with smaller values indicating a greater impact. The value <0.05 is considered to have a significant effect. **B** Counts of NHB-SHB subgroup-specific DMRs in three contexts: CG, CHG, and CHH. **C** The overlap between CG, CHG, and CHH hyper-DMRs (DMR hypermethylated in NHB, left) and hypo-DMRs (DMR hypomethylated in NHB, right). **D** The distribution of various types of DMRs among different genomic features (exon, intergenic region, intron, and transposon).

DNA methylation is important for maintaining genomic stability and is often considered a repressive epigenetic marker for transposable elements (TEs) and gene expression [38]. In the genome region with the most pronounced differentiation between the NHB and SHB subgroups, we identified two genes with sequence homology to Arabidopsis *RNA-DIRECTED DNA METHYLATION 1 (RDM1, AT3G22680)* and *DEMETER (DME, AT5G04560)*, which are involved in RNA-directed DNA methylation and active DNA demethylation, respectively [38]. We identified three missense variants in both *VcDME* and *VcRDM1* (Fig. 4A; Fig. S13, see online supplementary material). Compared to NHB, SHB tends to contain a higher proportion of heterozygous loci (Fig. 4A; Fig. S13, see online supplementary material). The results suggest a differential distribution frequency of distinct haplotypes in NHB and SHB cultivars for both genes. The allele distribution frequency of these missense variants was also analysed in NHB and SHB cultivars. Similar to our observation for *VcTBL44*, while *VcRDM1* in NHB cultivars all appeared to be homozygous, there seemed to be a correlation between the distribution frequency for all three missense variants at *VcRDM1* loci in SHB (Fig. S14, see online supplementary material). However, no strong association was observed for the allele distribution frequency at three *VcDME* variant loci (Fig. S15, see online supplementary material).

To further explore the possible differences in DNA methylation patterns between NHB and SHB subgroups, we conducted whole-genome bisulfite sequencing using leaf samples collected from 12 representative SHB and nine representative NHB cultivars grown naturally in the same habitat (Table S7, see online supplementary material). These cultivars were selected based on our population structure analysis showing their classification as typical SHB and NHB cultivars (Fig. 1A–C). A total of approximately 450 Gb of data was generated (Table S7, see online supplementary material). After mapping to the pseudo-reference genome and applying strict filtering (see Materials and methods), the average coverage and sequencing depth were 90.59% and 14.73-fold, respectively (Table S7, see online supplementary material).

The overall level of methylation was not significantly different between the two subgroups in all three sequence contexts (CG, CHG, and CHH, where H represents A, T, or C; Fig. S16A, see online supplementary material). Next, we identified subpopulation-specific differentially methylated regions (DMRs) between NHB and SHB, comprising 825 CG-DMRs, 1425 CHG-DMRs, and 1176 CHH-DMRs (Fig. 4B). While we observed a slightly greater number of NHB hyper-DMRs than NHB hypo-DMRs for CG and CHG DNA methylation, the difference was much greater in the CHH context (Fig. 4B). We assessed the length of the DMRs and found that CHG-DMRs were the longest, CG-DMRs were of intermediate length, and CHH-DMRs were the shortest (Fig. S16B, see online supplementary material). We also found that CHH-DMRs displayed minimal overlap with CG- and CHG-DMRs (Fig. 4C), implying that CHH-DMRs and CG/CHG-DMRs mainly occur in different regions. Further analysis revealed that CHH hyper-DMRs (DMRs hypermethylated in NHB) were more enriched in TE regions, whereas the latter two (CG- and CHG-DMRs) were mainly found in intergenic and genic regions, including exons and introns (Fig. 4D; Fig. S16C, see online supplementary material). To summarize, our results suggest that while the overall level of DNA methylation is similar between NHB and SHB cultivars, thousands of sites are differentially methylated between the two subgroups. Moreover, DNA methylation of different sequence contexts was associated with different genomic elements, with CHH-DMRs showing a higher correlation with TEs.

### Genes in the β-alanine metabolism pathway are differentially methylated between NHB and SHB cultivars

**Figure 5:**
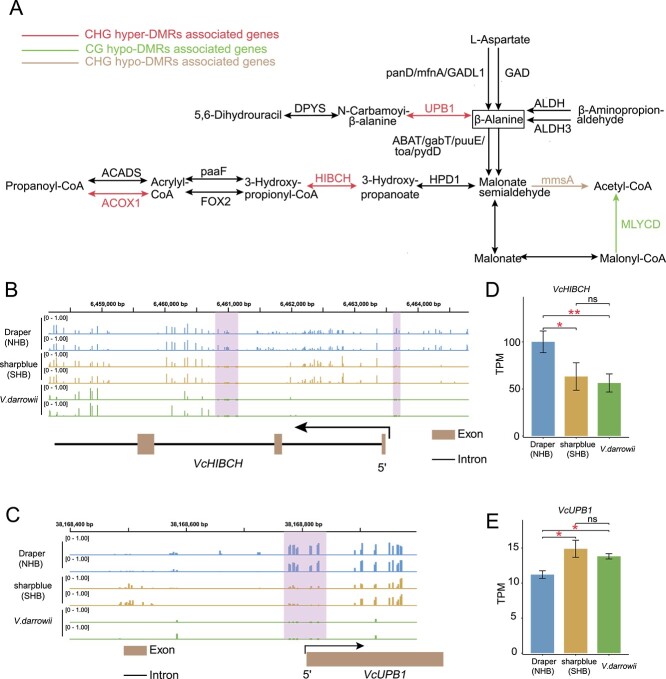
The association between DNA methylation and the β-alanine metabolism pathway. (**A**) The pathway is significantly enriched for genes overlapping with DMRs. Arrows of different colors (except black) represent the influence of different types of DMRs. (**B**) Genome browser view showing the level of CHG methylation of *VcHIBCH* in ‘Draper’ (blue), ‘sharpblue’ (orange), and *V. darrowii* (green). Pink shading represents DMRs. (**C**) Same as in (**B**), except for *VcUPB1*. (**D**) Expression level of *VcHIBCH* in ‘Draper’ (blue), ‘sharpblue’ (orange), and *V. darrowii* (green). TPM (transcripts per kilobase per million mapped reads) represents the expression level. Statistically significant differences between the groups were assessed using Student's *t*-test. ‘ns’ indicates no significant difference. Significant results are marked by one red asterisk (*P*-value <0.05) and two red asterisks (*P*-value <0.01). **E** Same as in (**D**), except for *VcUPB*.

Next, we focused on functional genes that were differentially methylated between NHB and SHB subgroups. We found that 2042 genes were associated with NHB-SHB DMRs. Among them, 1416 genes were associated with NHB hyper-DMRs, and 698 genes were associated with NHB hypo-DMRs (Table S8, see online supplementary material). We conducted a Kyoto Encyclopedia of Genes and Genomes (KEGG) enrichment analysis on these differentially methylated genes and observed the enrichment of biological pathways, including the β-alanine metabolism pathway, the environmental information processing, and metabolism of terpenoids and polyketides, etc. (Fig. S17, see online supplementary material). Previous studies demonstrated the role of increased β-alanine accumulation in promoting plant resistance to heat and drought stresses [39, 40]. To gain a deeper understanding of the impact of DNA methylation on β-alanine metabolism, we re-constructed the core β-alanine metabolism network (Fig. 5A) and discovered that three genes within this network are associated with NHB hyper-DMRs in the CHG context (Fig. 5A; Fig. S18, see online supplementary material), whereas one gene was associated with NHB CG hypo-DMRs (Fig. 5A).

Next, we wanted to ascertain whether differential methylation of genes in the β-alanine metabolism pathway was also associated with differential gene expression. Transcriptome analysis was conducted using leaf samples collected from three representative cultivars showing typical DNA methylation patterns for NHB, SHB, and *V. darrowii*, respectively (see Materials and methods; Fig. S19, see online supplementary material). SHB and *V. darrowii* displayed similar levels of DNA methylation of *VcHIBCH* and *VcUPB1* compared to NHB, and the gene expression levels reflected this pattern (Fig. 5B–E). Similarly, examination of other genes in the β-alanine metabolism pathway, such as *VcALDH*, *VcACOX1*, and *VcGAD*, also revealed gene expression and methylation levels more similar between SHB and *V. darrowii* than with NHB (Fig. S20, see online supplementary material). Our discovery is aligned with the notion that *V. darrowii* has been used as a genetic material donor to improve the adaptability of SHB to warmer climates.

### Characterization of TE variations between the NHB and SHB subgroups

**Figure 6:**
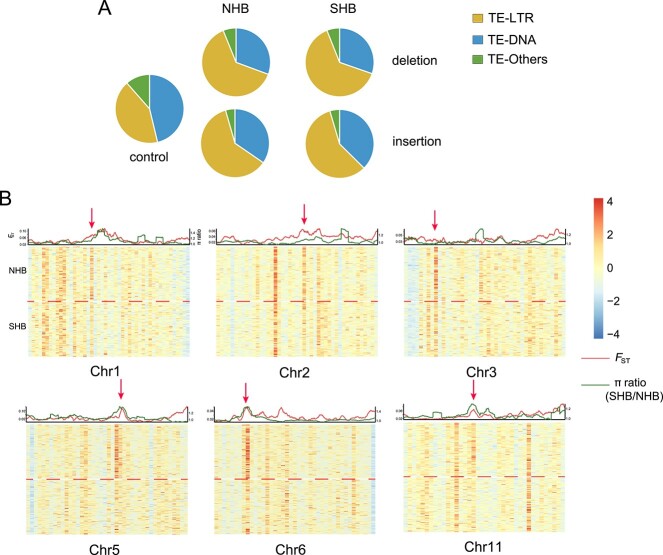
TE variations among NHB and SHB subgroups. **A** The distribution of TE deletions (upper) and TE insertions (lower) in the NHB (left) and SHB (right) subgroups. **B** Distribution of the density of TE insertions across chromosomes 1, 2, 3, 5, 6, and 11, with the distribution of *F*_ST_ values (red curve) and π ratios (πSHB/πNHB, green curve) displayed above the heatmap. Red arrows indicate TE variation sites.

In addition to regulating gene expression, DNA methylation also plays an important role in suppressing the activity of transposons to maintain genome integrity [38]. Therefore, we aimed to understand if the population differentiation between NHB and SHB is also associated with differential TE insertion or deletions between NHB and SHB. To answer this question, we conducted a whole-genome analysis of transposon variations in 83 SHB and 81 NHB accessions. Compared to other transposons, we found that LTR-RTs exhibited greater activity, showing a higher frequency of insertion and deletion variations compared to other types of TEs (Fig. 6A). We further divided the genome into 50-kb windows and assessed the density of TE variations within each window for both insertion and deletion events. Comparison between NHB and SHB showed an enrichment of TE insertions in NHB relative to SHB at several genomic loci (chromosomes 1, 2, 3, 5, 6, 11, 12; Fig. 6B; Fig. S21, see online supplementary material). Interestingly, several of these loci differentially enriched in TE variation also overlapped with regions displaying high population differentiation, as indicated by high *F*_ST_ and π ratio (SHB/NHB) (Fig. 6B; Fig. S21, see online supplementary material). In contrast, no differential enrichment of TE deletions was observed between NHB and SHB (Fig. S22, see online supplementary material). Additionally, we found no evidence that these NHB-SHB TE variations are associated with differential DNA methylation. These findings indicated that transposon variations, especially differential insertions between NHB and SHB, may be involved in population differentiation between the two blueberry subgroups.

## Discussion

Blueberry genomes exhibit complex ploidy, with diploid, tetraploid, and hexaploidy varieties present [1]. Several reference genomes for blueberry have been published, including the tetraploid NHB cultivar ‘Draper’ (*V. corymbosum*) [12], diploid *V. darrowii* [13, 14], the diploid wild blueberry, *V. bracteatum* [41], and the most recent blueberry pan-genomes covering blueberry and cranberry [17]. We assessed the collinearity between the ‘Draper’ genome and the *V. darrowii* genome [13] and found that while the genomes exhibit considerable consistency, they also have extensive large-scale variations such as inversions, translocations, and duplications (Fig. S23, see online supplementary material). Additionally, because the majority of the materials in our population were tetraploid *V. corymbosum* (including NHB and SHB, Table S1, see online supplementary material), we opted to use the ‘Draper’ as the reference genome. Following the convention of other previously published studies, the longest scaffolds of each homologous chromosomes (a total of 12 scaffolds) were selected as reference genomes for mapping [5, 15, 42].

Analysing species with complex ploidy at the population level faces several challenges, as variations in ploidy levels among individuals may introduce bias in alignment and allelic dosage effects. In our case, a diploid model was applied to facilitate the adaptation of our datasets to existing analytical approaches, a strategy also used in alfalfa [28] and sugarcane [43]. Previous studies have shown that simplifying into diploid models has minimal impact on the analysis of population structure [5]. To evaluate the potential impact of simplifying polyploids into diploids on our study’s conclusions, we compared the contribution of the top 10 principles between strategies using the diploid and continuous (polyploid) models, following the approach outlined by Soichiro Nishiyama [5]. Specifically, we selected 10 tetraploid SHB accessions, 10 tetraploid NHB accessions, and 10 hexaploid RB accessions. Genomic variations were identified using both diploid and continuous models. Subsequently, we computed the first 10 principal components for each model separately. We found that in both models, each principal component exhibits highly significant correlations (Fig. S24A, see online supplementary material), and these 10 principal components can account for most of the variation in the blueberry population (Fig. S24B, see online supplementary material). Furthermore, using the first two principal components, both models displayed similar population distributions (Fig. S24C, see online supplementary material). Collectively, the analysis has indicated that simplifying polyploids as diploids had minimal impact on our interpretation of the blueberry population properties. To further minimize the impact of ploidy simplification, we focused our efforts on the comparison between the NHB and SHB subgroups, which are predominantly composed of tetraploid cultivars (Table S1, see online supplementary material). Although this simplified model has been successfully applied in calculating population structure and gene flow, this method can lead to an underestimation of genetic diversity [28, 43]. The more accurate analysis results await the development of more advanced techniques and algorithms.

We presented the population structure and evolutionary relationships of the major cultivated blueberry subgroups. Overall, the differentiation among subgroups was not particularly pronounced, and there was extensive gene flow between the two main cultivated blueberry subgroups, NHB and SHB. Notably, we also found potential genetic introgression from other species, especially lowbush blueberry (LB), into NHB (Fig. 1C). Therefore, we carried out gene introgression analysis for NHB, and examined potential gene flow from LB, *V. darrowii*, and *V. ashei* (Fig. S25A–C, see online supplementary material). The results showed a significant amount of genetic introgression from LB into NHB (Fig. S25A, see online supplementary material), which is consistent with the known breeding history of NHB. GO analysis revealed that genes in the introgression region were mainly associated with plant root morphogenesis, endoderm development, and flower and reproductive development (Fig. S25D, see online supplementary material). In contrast, the extent of gene introgression from *V. darrowii* and *V. ashei* into NHB is much less prominent (Fig. S25B–C, see online supplementary material). Previous studies of blueberry population genetics revealed that the two main cultivated blueberry subgroups, NHB and SHB, are genetically closely related [5, 15, 24]. Consistent with this finding, we also observed that NHB and SHB do not form distinct and separate lineage branches (Fig. 1A). In addition, we employed whole-genome resequencing at 35-fold coverage to generate a high-density SNP map. The high-density SNP map not only allowed for a more accurate dissection of the blueberry population structure but also facilitated the characterization of the genetic divergence between NHB and SHB. By characterizing regions exhibiting clear population differentiation between the NHB and SHB subgroups, we found that the SHB subgroup tended to exhibit higher genetic diversity in the differentiated region. This higher diversity is likely attributed to gene introgression from *V. darrowii* and *V. ashei* (Fig. 2C). Treating polyploids as diploids in the calculation of f_d_ may overlook the dosage effects of alleles, leading to an underestimation of genetic diversity and the level of genetic introgression [5, 28, 43]. For example, autotetraploids with genotypes ‘AAAB’, ‘AABB’, and ‘ABBB’ are all recognized as ‘AB’. Therefore, the genetic introgression among these three genotypes cannot be identified, increasing the threshold of counting f_d_ values. In our analysis we likely underestimated the level of genetic introgression from *V. darrowii* and *V. ashei* into SHB.

Moreover, our findings indicate that the introgression of key functional genes, such as *VcHSP70* and *VcPARK13* has probably contributed to the improved subtropical adaptation and fruit development in SHB cultivars, respectively. The importance of *VcTBL44* was not only supported by its differential expression during fruit development, but also by its appearance as a GWAS candidate gene (Fig. 3B). However, due to the differential acclimatization of NHB and SHB subgroups, and the divergence in developmental and fruit-setting traits they exhibit, it is challenging to conduct phenotypical measurements simultaneously for large numbers of NHB and SHB cultivars in the same field. Therefore, the GWAS conducted in this study involved a limited number of cultivars (*n* = 50). Further experimental validations are required to conclude the functional importance of the GWAS candidate genes.

DNA methylation is a well-conserved epigenetic modification that has been shown to contribute significantly to crop domestication and improvement [18, 22, 44]. Comparison of NHB and SHB cultivars revealed a significantly greater number of NHB CHH hyper-DMRs than CHH hypo-DMRs (1132 vs 44, Fig. 4B). Further investigation showed that these NHB CHH hyper-DMRs are mainly associated with transposons over other types of genomic features (Fig. 4D). We also identified several genomic loci (chromosomes 1, 2, 3, 5, 6, 11, 12) that exhibited an enrichment of TE insertions in NHB compared to SHB (Fig. 6B; Fig. S21, see online supplementary material). These NHB-specific TE insertions could possibly lead to *de novo* CHH methylation as a means to suppress TE activity and maintain genome integrity. Alternatively, the lack of TE insertions and associated CHH methylation in SHB relative to NHB may be attributed to interspecific hybridization and reflect genomic features derived from *V. darrowii* and possibly other parental species used in development of SHB cultivars. Analysis using *V. darrowii* combined with long-read sequencing will provide better answers to this question in the future.

## Materials and methods

### Plant materials and genome resequencing

A total of 219 accessions belonging to the *Vaccinium* genus, including 81 NHBs, 83 SHBs, seven HHBs, 15 RBs, 10 LBs, 12 CBs, and 11 other related species were collected from Changchun Experimental Station of National Germplasm Resources, Ministry of Agriculture and Rural Affairs, College of Horticulture, Jilin Agricultural University of China (43°80′N, 125°42′E; Table S1, see online supplementary material). One accession of *V. darrowii* was collected from Institute of Botany, Jiangsu Province and the Chinese Academy of Sciences (31°56′N, 118°45′E; Table S1, see online supplementary material).

The CTAB method was employed to extract genomic DNA from leaves [45]. For each sample, 0.2 μg of DNA was used as input material for DNA library preparations using the NEB Next® Ultra™ DNA Library Prep Kit for Illumina (NEB, in Ipswich, MA, USA), following the manufacturer’s instructions. Genomic DNA samples were fragmented by sonication to a size of 350 bp. Sequencing indexes were then added to each sample to generate the sequencing library. Clustering of the indexed samples was performed on a cBot Cluster Generation System using the Illumina PE Cluster Kit (Illumina, in San Diego, California, USA) according to the manufacturer’s instructions. After cluster generation, the DNA libraries were sequenced on an Illumina NovaSeq 6000 platform to generate 150-bp paired-end reads.

### Phenotyping

For phenotyping, 50 blueberry cultivars (including 18 NHB cultivars and 32 SHB cultivars) were sampled from the Joint International Research Laboratory of Modern Agricultural Technology College of Horticulture, Jilin Agricultural University of China (43°80′N, 125°42′E) in 2021. Each cultivar was grown in a designated space of the same growth facility. To measure the fruit phenotype, 10 berries at the full purple stage were harvested for each cultivar, with each fruit treated as a single replicate. We only sampled berries exhibiting picking quality, including fully blue color, and no visible pathogen or insect damage.

The samples were deformed to 30% of the original height using a crosshead speed of 2 mm/s and a 75-mm diameter cylinder stainless flat probe in a Texture Analyser (TMS-PRO, in Atlanta, GA, USA) [46]. The texture profile analysis was performed with a starting position of the probe of 20 mm from the platform surface. The probe started moving at a pretest speed of 5 mm/s until it touched the blueberry, which was indicated by reaching a trigger force of 0.2 N. At that point, the probe initiated the first compression (downstroke) at a test speed of 2 mm/s until the target strain of 20% of the blueberry’s equatorial height was achieved.

After reaching the chosen deformation distance, the probe ascended (upstroke) at a test speed of 2 mm/s to the position where it first recognized the trigger force for the initial compression. At the end of the selected waiting time, the probe began a second compression, descending to the same target distance at the same test speed as the first compression. Finally, the probe ascended to the starting position at a post-test speed of 1.66 mm/s. Based on the force-time curve, the following five fruit characteristics were calculated: (i) firmness was the peak force of the first compression cycle; (ii) cohesiveness was measured by the area of work during the second compression divided by the area of work during the first compression; (iii) gumminess was calculated as firmness × cohesiveness; (iv) chewiness was calculated as gumminess × springiness; and (v) springiness was measured as the distance of the detected height of the product during the second compression. Experimental data were presented as mean ± standard deviation (Table S4, see online supplementary material). For each cultivar, the measurements were repeated 10 times and then averaged.

### Sequence alignment and variant calling

First, the raw data were filtered using Fastp v0.20.1 with the default parameters [47]. Subsequently, the reads from all accessions were mapped to the largest genome scaffolds of each of the 12 homoeologous groups (Scaffolds 1, 2, 4, 6, 7, 11, 12, 13, 17, 20, 21, and 22, representing chromosomes 1–12) of the ‘Draper’ cultivar of *V. corymbosum* using BWA-MEM [48]. The scaffold numbering also follows the convention of the original article [12]. Only the reads that were uniquely mapped were kept. The Java program MarkDuplicates.jar from the Picard Toolkit v2.25.6 (Picard Toolkit. 2019. Broad Institute, GitHub Repository. https://broadinstitute.github.io/picard/; Broad Institute) was used to remove PCR duplicates.

Following read mapping, we utilized SAMtools v1.12 [49] and BCFtools v1.8 [50] to call raw genomic variants, including SNPs and indels. Specifically, we used the ‘mpileup’ option in SAMtools v1.12 to generate a bcf file that contained variant information. The resulting bcf file was then converted to the VCF format using BCFtools v1.8. To obtain high-quality SNPs, we filtered the VCF file using VCFtools v0.1.16 [51], with the criteria of minor allele frequency (MAF) >5%, missing data rate <10%, minimum quality score >30, sequencing depth >4 and <100. We further annotated the SNPs based on their genomic locations and predicted coding effects using SnpEff v5.1 [52], using GFF files (the annotation file of all coding regions of each gene) derived from the genome sequence of the ‘Draper’ cultivar of *V. corymbosum*.

### Ploidy prediction

The genomic ploidy level was predicted using nQuire [23]. For each fixed model, log-likelihood values were computed based on the denoised base frequency distribution. The fixed model exhibiting the lowest Δlog-likelihood value compared to the free model was chosen as the predicted ploidy level.

### Population structure analysis

To perform population structure analysis, we further filtered high-quality SNPs (after filtering) in LD using PLINK v1.90b6.21 [53] with a window size of 50 SNPs (advancing 10 SNPs at a time) and an r^2^ threshold of 0.1. The remaining 609 456 high-quality SNPs in LD were used to construct the evolutionary tree using FastTree v2.1.10 [54] with the maximum likelihood method. We performed 1000 bootstraps to assess the robustness of the tree. The resulting tree was visualized and colored using the iTOL tool (http://itol.embl.de) [55].

We performed the PCA using PLINK v1.90b6.21 [53] with default settings. The first two eigenvectors were retained to create a plot using R. Population structure was analysed using the ADMIXTURE v1.3.0 program [56] with the default parameters.

For testing the diploid and continuous models on small samples. VCF files were generated using SAMtools v1.12 and VarScan v2.4.6 [49, 57], followed by filtering as described earlier. PCA was then performed using the R package pcaMethods [58].

### Detection of population differentiation and gene introgression

We calculated nucleotide diversity (π) and genetic differentiation (*F*_ST_ values) between NHB and SHB using VCFtools v0.1.16 [51] with 50-kb windows sliding in 5-kb steps. To identify regions with evident differentiation between NHB and SHB, we selected windows with significantly high *F*_ST_ values (the 5% right tail) and significantly low and high π ratios (the 5% left and right tails). These intervals were thought to demonstrate notable population differentiation and disparities in nucleotide diversity between NHB and SHB.

The f_d_ value was employed to assess potential introgression from *V. darrowii* and *V. ashei* into the SHB subgroup [29]. Since H82 (‘Pink Lemonade’, ARS 96–138) has mixed genetic background (Fig. 1C), we have excluded this accession from the gene introgression analysis. *V. macrocarpon* (O) was used to infer ancestral states in the *V. darrowii/V. ashei* (P3), NHB (P1), and SHB (P2) populations. Without gene flow, the ABBA and BABA allele configurations in the tree (((P1, P2), P3), O), should be equally frequent. If gene flow occurred between *V. darrowii/V. ashei* and the SHB subgroup, the value of ABBA relative to BABA would increase. The f_d_ statistic was computed in 50-kb sliding windows with a 5-kb step [29]. Windows containing fewer than five informative SNPs were disregarded, and windows with negative Patterson’s D statistic values and f_d_ > 1 were also excluded. The top 1% of intervals with the highest f_d_ values were designated as strong introgression regions, and these were merged using bedtools v2.30.0 [59]. Blueberry candidate genes, such as *VcWRKY34*, *VcAP2*, *VcFCA*, *VcHSP70*, *VcPARK13*, and *VcLOX1/5* were named from their closest homologs in Arabidopsis.

### Whole-genome bisulfite sequencing (WGBS) and bioinformatics analysis

We collected leaf samples (12 accessions of SHB and nine accessions of NHB) from 8-year-old plants in July 2021 from Changchun Experimental Station of National Germplasm Resources at Jilin Agricultural University of China (43°80′N, 125°42′E). Each cultivar was planted in a designated area, with one replicate collected for each cultivar. The ‘sharpblue’, ‘Draper’, and *V. darrowii* samples used for DNA methylation sequencing were also collected from leaf samples sourced from the Institute of Botany, Jiangsu Province and the Chinese Academy of Sciences (31°56′N, 118°45′E). Two replicates were taken for each cultivar.

Genomic DNA was extracted from leaves using the CTAB method [45]. To prepare WGBS libraries, the EpiArt DNA Methylation Library Kit for Illumina V3 was utilized following the manufacturer’s protocol. The EpiArt DNA Methylation Bisulfite Kit was employed to perform bisulfite conversion. The resulting DNA libraries were sequenced on the Illumina NovaSeq 6000 platform.

The analysis of WGBS data involved several steps. First, adaptors were trimmed from the raw reads using Fastp v0.20.1 [47]. To reduce the impact of single nucleotide variations in each accession on the alignment, we replaced the homozygous nucleotide variations in each accession with the reference genome allele to generate a pseudo-reference genome. In the case of heterozygous loci, where one allele is identical to the ‘Draper’ reference genome and the other allele is different, we retained the allele that is identical to the reference genome for downstream analysis. Then, the clean reads were aligned to the pseudo-reference genome using BS-Seeker2 v2.1.8 [60]. The following settings were used: —aligner = bowtie2, —bt2—end-to-end, −m = 0.08, —XSteve. Alignments with a Phred score (Q) <30 were removed using SAMtools v1.12 [49]. PCR duplicates were removed using a Java program called MarkDuplicates.jar from the Picard Toolkit V2.25.6 (Picard Toolkit. 2019. Broad Institute, GitHub Repository. https://broadinstitute.github.io/picard/; Broad Institute). Methylation levels were then calculated using CGmapTools v 0.1.2 [61]. DMRs were identified using Metilene v0.2–8 [62], with the requirement that a DMR must contain at least eight cytosine sites and have a q-value <0.01 (Bonferroni). For CG, CHG, and CHH methylation, the difference between two samples was considered significant if it was greater than 0.2, 0.2, and 0.1, respectively. Finally, DMR-associated genes were defined as those containing DMRs within 2 kb.

### RNA sequencing and bioinformatics analysis

We collected leaves from the ‘sharpblue’, ‘Draper’, and *V. darrowii* accessions from the Institute of Botany, Jiangsu Province and the Chinese Academy of Sciences (31°56′N, 118°45′E) for transcriptome sequencing, with three replicates for each cultivar. Each sample was planted in the same greenhouse, which suited their growth requirements, to ensure normal development. Total RNA from leaves of each cultivar was extracted using the Plant RNA Extraction Kit and subsequently employed to construct cDNA libraries with a fragment length of 300 bp. The cDNA libraries were sequenced on the Illumina NovaSeq 6000 platform. For transcriptome analysis during fruit development, samples were collected at five different stages based on changes in fruit size and external color (Fig. S8, see online supplementary material), including SG, MG, LG, PP, FP. Three replicates were collected, with each replicate containing six fruits. Total RNA from fruits was isolated from the strain using the Trizol reagent according to manufacturer’s instructions (Invitrogen) and sent to Novogene (Tianjin, China) for library preparation and deep sequencing.

For RNA-seq analysis, adaptors were trimmed from the raw reads using Fastp v0.20.1 [47]. The trimmed reads were aligned to the pseudo-reference genome and ‘Draper’ reference genome using HISAT2 v2.2.1 under default settings [63]. The expression level of each gene, measured as TPM (transcripts per kilobase of exon model per million mapped reads) values, were calculated using featureCounts of the Subread package v2.0.2 with default parameters [64]. Differentially expressed genes were defined by DESeq2 using |log_2_FC| > 1 and *P*.adj < 0.05 as cut-offs [65].

### Enrichment analysis

GO enrichment analysis was conducted on the genes in differentially selected regions using the clusterProfiler package v4.4.4 [66]. The genes associated with DMRs were subjected to KEGG enrichment analysis using TBtools v1.105 [67]. Significant enrichment was determined using the Benjamini & Hochberg method with a corrected *P*-value threshold of <0.05.

### GWAS of fruit firmness

Utilizing high-quality SNPs, we conducted a GWAS analysis using a linear mixed model implemented by the EMMAX package [68]. We performed principal component analysis and used the first two principal components (PC1 and PC2) for correction. A genome-wide significance cutoff of 1e-5 was chosen. Subsequently, we expanded the candidate region to 10 kb around the peak of the GWAS signal to identify candidate genes.

### Assessment of deleterious variations

We predicted the effects of missense SNPs on protein function using the SIFT algorithm [37]. If the SIFT score was <0.05, the SNP was considered to have a significant impact on protein function.

### Phylogenetic analysis of *TBL* homologs

Protein sequences of *TBLs* from *Arabidopsis thaliana* were obtained from the TAIR database (https://www.arabidopsis.org). Candidate genes in blueberries were identified using blastp v2.5.0 [69] with the following filtering thresholds: sequence identity >30%, e-value <1e-10, and a score >200. Multiple sequence alignment was performed using Muscle v3.8.1551 [70], and the resulting alignment was trimmed using Trimal v1.4.rev15 [71]. A phylogenetic tree was constructed using the maximum likelihood method in IQ-TREE v2.0.3 with 1000 bootstraps [72].

### Transposable element (TE) variant detection

To detect transposon variations, we re-annotated the transposons in the Draper’ genome using EDTA v2.0.1 [73] and further classified the unknown categories using DeepTE [74]. TE variants were detected using TEPID v0.10 [75]. For paired-end resequencing data, TEPID initially employed yaha for single-end alignment followed by bowtie2 for paired-end alignment [76, 77]; both rounds of alignment were compared to identify instances of abnormal alignment. By comparing the positions of aberrant alignments with the TE annotations in the reference genome, we identified transposon insertion and deletion variations. The average insert size was set to 350, and all other parameters were set as default.

## Supplementary Material

Web_Material_uhae138

## Data Availability

Whole-genome resequencing data of 220 blueberry accessions has been deposited into the Sequence Read Archive (SRA) database under accession number PRJNA948809. The whole-genome methylation data of 21 cultivars (including 11SHB and 10 NHB), as well as the whole-genome methylation and transcriptome data of ‘Draper’, ‘sharpblue’, and *V. darrowii*, have been deposited into the Gene Expression Omnibus (GEO) database under SuperSeries GSE228041 (https://www.ncbi.nlm.nih.gov/geo/query/acc.cgi?acc=GSE228041) . RNA-seq data of five developmental stages of blueberry have been deposited into the Gene Expression Omnibus (GEO) database under SuperSeries GSE229139. SNPs and indels in Variant Call Format (VCF) have been deposited into the Figshare database (https://doi.org/10.6084/m9.figshare.22699810).
