# Supplementary material for: Genetic and epigenetic signatures for improved breeding of cultivated blueberry
Source: Hortic Res. 2024 May 14;11(7):uhae138. doi: 10.1093/hr/uhae138 (PMC11233858; doi:10.1093/hr/uhae138)
Supplement: Web_Material_uhae138 [file web_material_uhae138.zip › Supplemental Figure.docx]

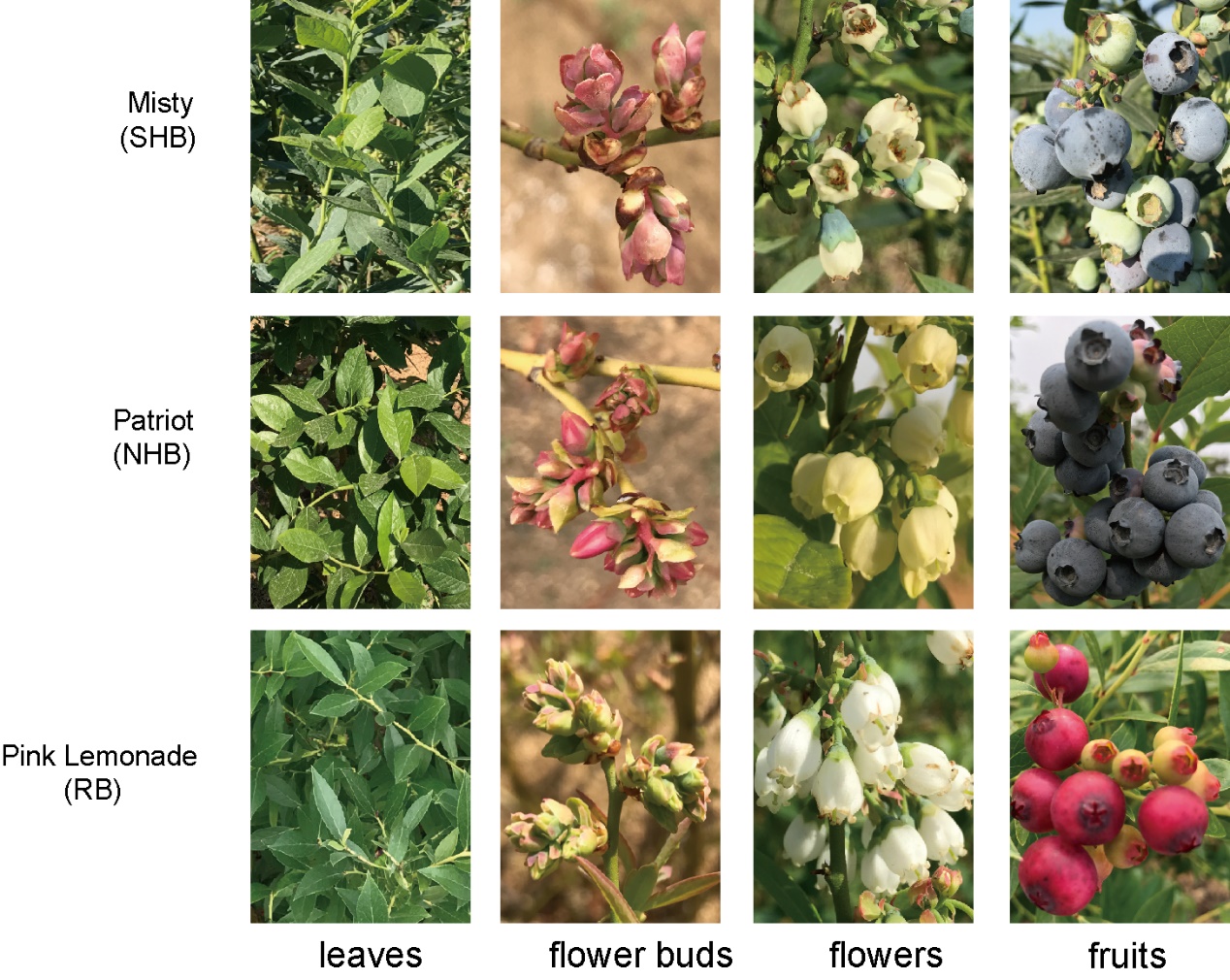


**Figure S1** Representative pictures showing leaves, flower buds, flowers, and fruits of three different blueberry cultivars (top: Misty; middle: Patriot; bottom: Pink Lemonade).


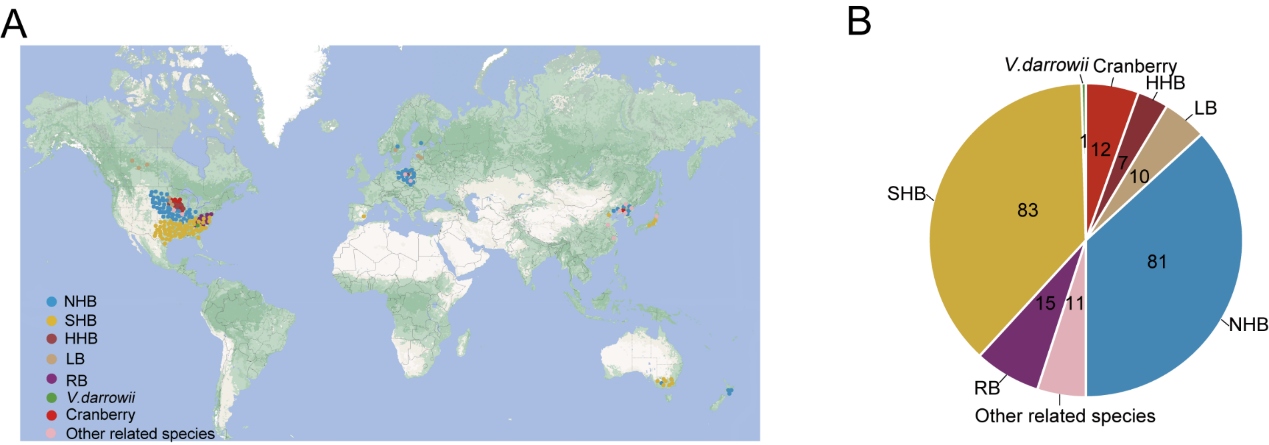


**Figure S2 The origin and composition of the population. (A)** The geographic distributions of core germplasm resources collected in this study. The positions of the dots represent country origins (not cities), and the colors of the dots represent different blueberry subgroups. The map was downloaded from Google (<https://www.google.com/maps>). **(B)** The composition of blueberry subgroups collected in this study. RB: rabbiteye blueberry; LB: lowbush blueberry; NHB: northern highbush blueberry; SHB: southern highbush blueberry; HHB: half-high blueberry.


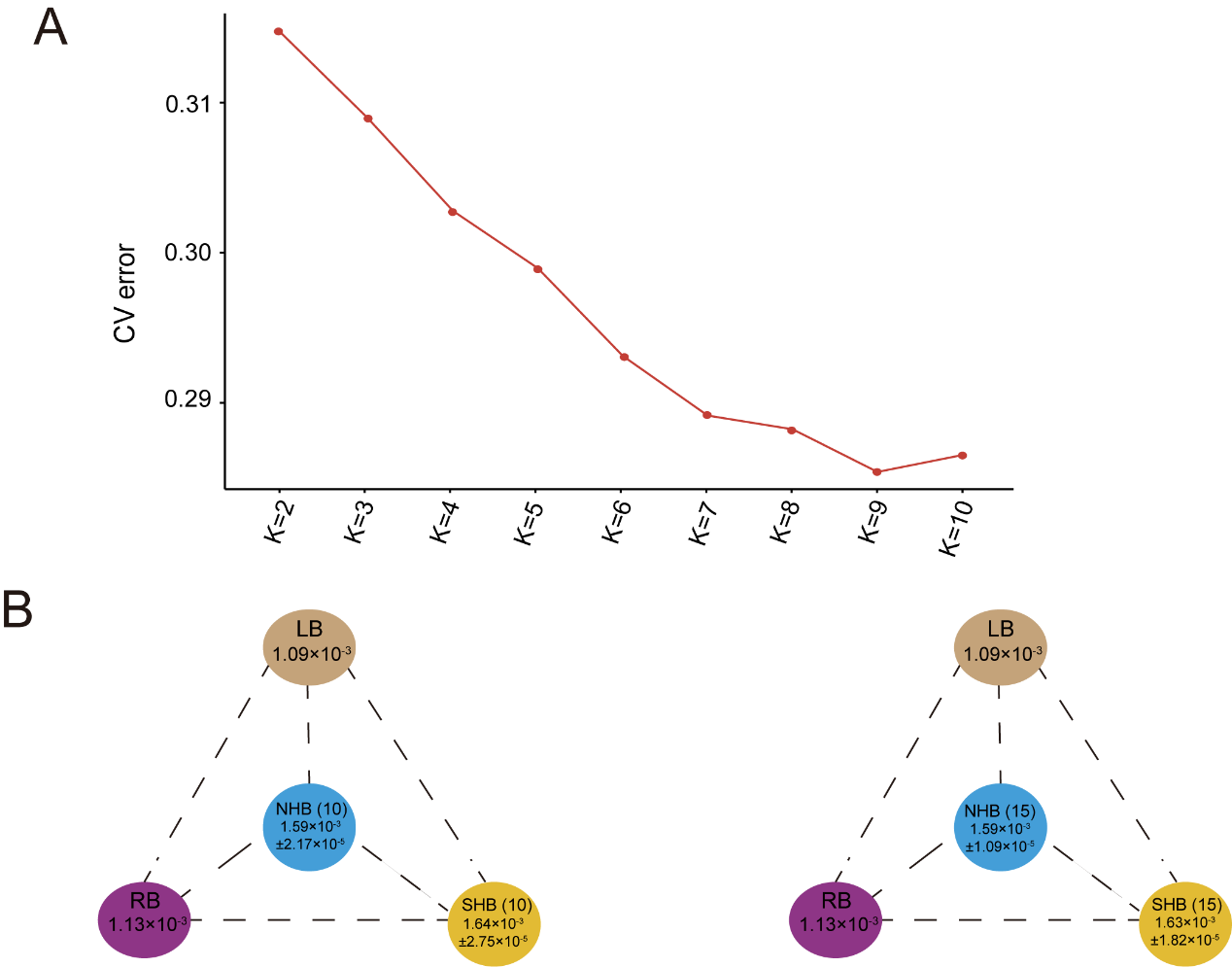


**Figure S3 Genetic analysis of cultivated blueberry. (A)** CV errors for the STRUCTURE analysis (K=2-10). **(B)** Nucleotide diversity (π) across four cultivated blueberry subgroups (calculated from the diploid model). The values in the circles represent the nucleotide diversity (π) of the groups (brown, orange, blue, and purple circles represent the LB, SHB, NHB, and RB subgroups, respectively). For NHB and SHB, the sample size was adjusted to 10 (left) and 15 (right), respectively, with each adjustment repeated 10 times.


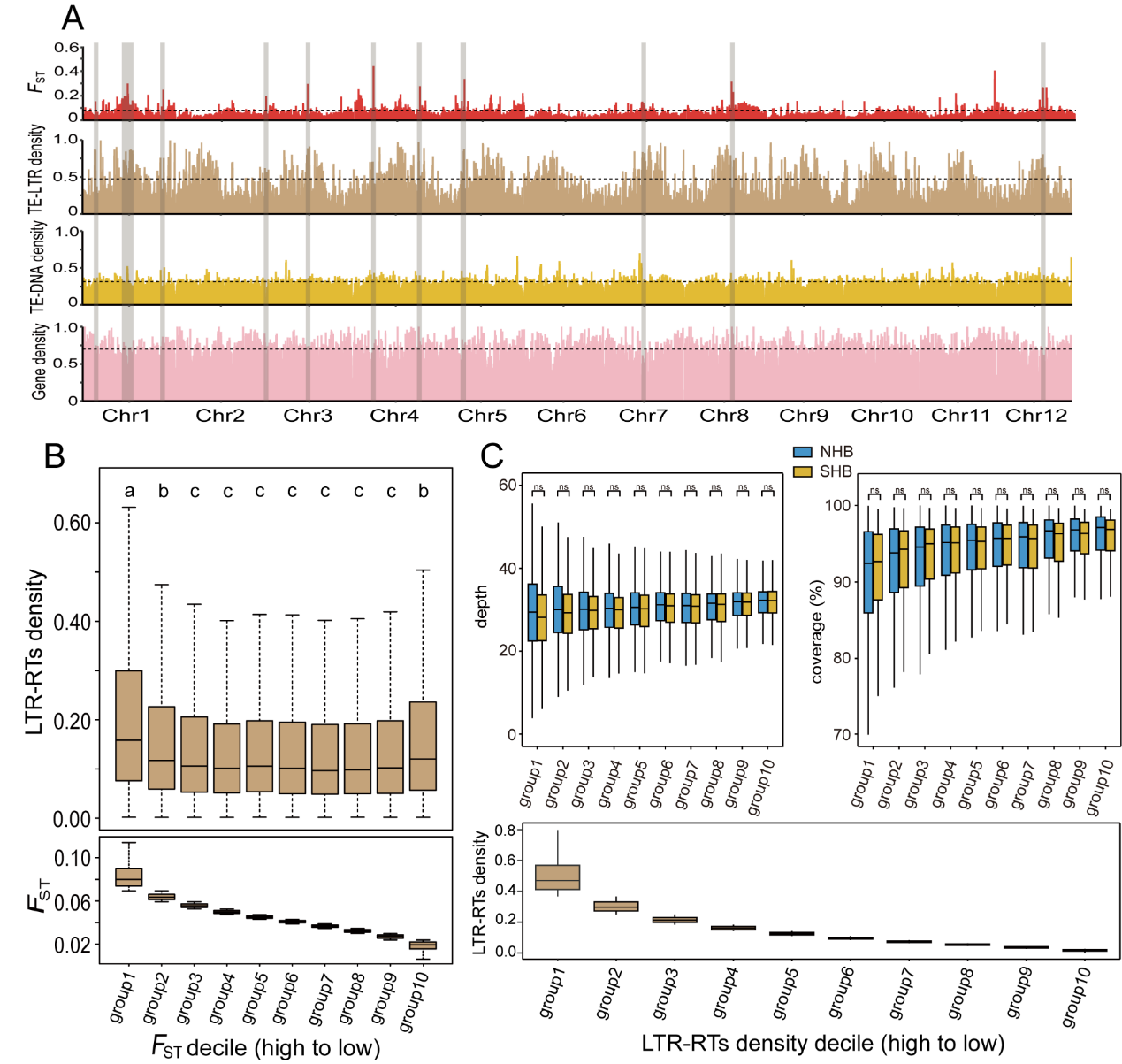


**Figure S4 The relationship between genetic differentiation and LTR-RTs. (A)** Genome-wide distributions of *F*_ST_ values (red bar), density of LTR-retrotransposons (brown bar), density of DNA transposons (orange bar), and density of genes (pink bar). The values above the black dashed line represent the top 5% of values.  **(B)** Boxplots showing density of LTR retrotransposons (upper panel) and *F*_ST_ values (lower panel) in different genomic regions. The blueberry genomic regions are divided into ten deciles based on their *F*_ST_ values, arranged from high to low. Different letters above the boxes indicate significant differences (*p*-value < 0.05, Bonferroni correction) in multiple comparison testing. **(C)** Comparison of alignment depth and coverage between NHB and SHB across different genomic deciles grouped by LTR-retrotransposon density. The below boxplots show the density of LTR-retrotransposons. Blueberry genomic regions are divided into ten deciles based on LTR-retrotransposon density, arranged from high to low. The upper boxplots display the comparisons of depth and coverage between NHB and SHB for each decile. "ns" is used to indicate no significant difference (Mann–Whitney–Wilcoxon test, two sided).


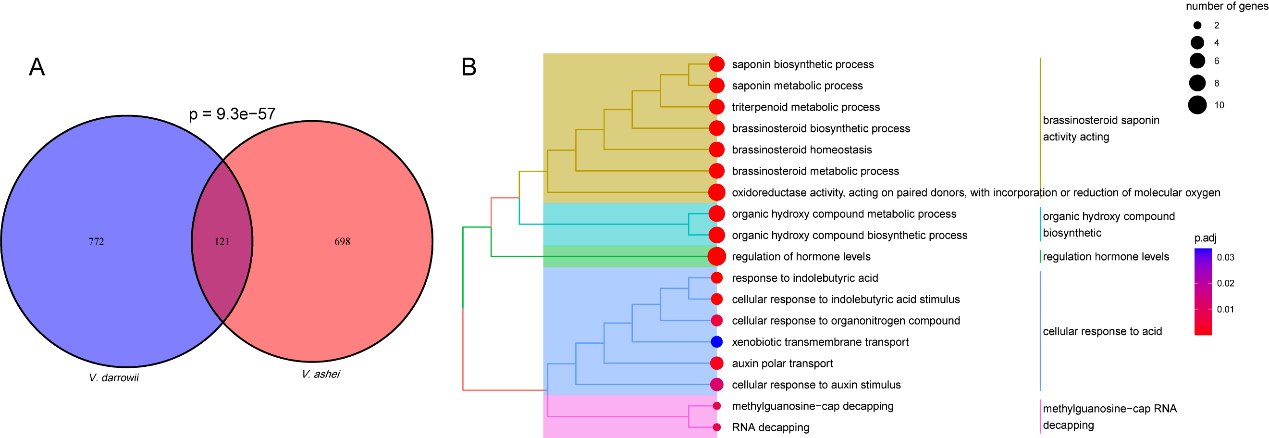


**Figure S5 Gene introgression from *V. darrowii* and *V. ashei* into SHB*.* (A)** The overlap of genes in the strong introgression regions (top 1%) of *V. darrowii* (blue) and *V. ashei* (red). Size is proportional to the number of genes defined in each group. The *p*-value indicates a statistically significant difference in the overlap (hypergeometric test, one sided). **(B)** Gene ontology (GO) enrichment of the common genes from both *V. darrowii* and *V. ashei* that were introgressed into the SHB subgroup region. The size of each dot represents the number of corresponding genes, while the color of the dot indicates the *p*-value associated with each GO term. GO terms are clustered using similarity indices.


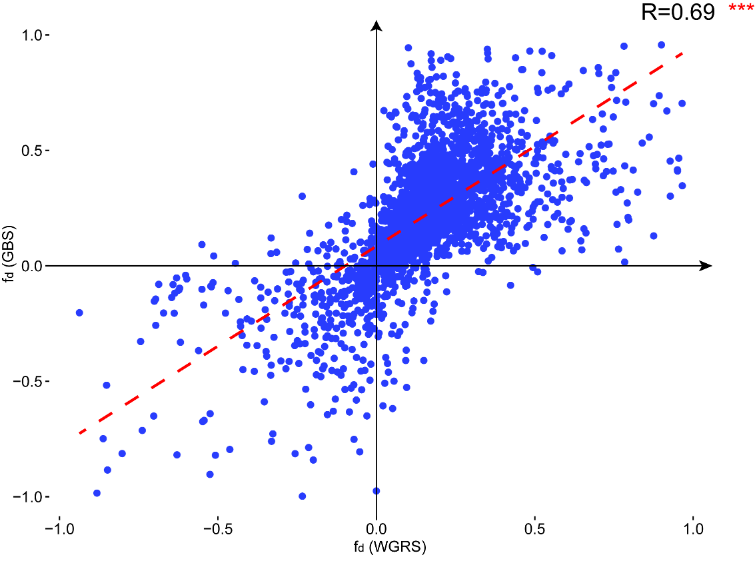


**Figure S6** The correlation between the f_d_ calculated from three accessions of *V. darrowii* (WGRS) and the f_d_ calculated from genotyping-by-sequencing (GBS) data of 81 *V. darrowii* by Manzanero et al. "***" represents the *p*-value less than 0.001.

**
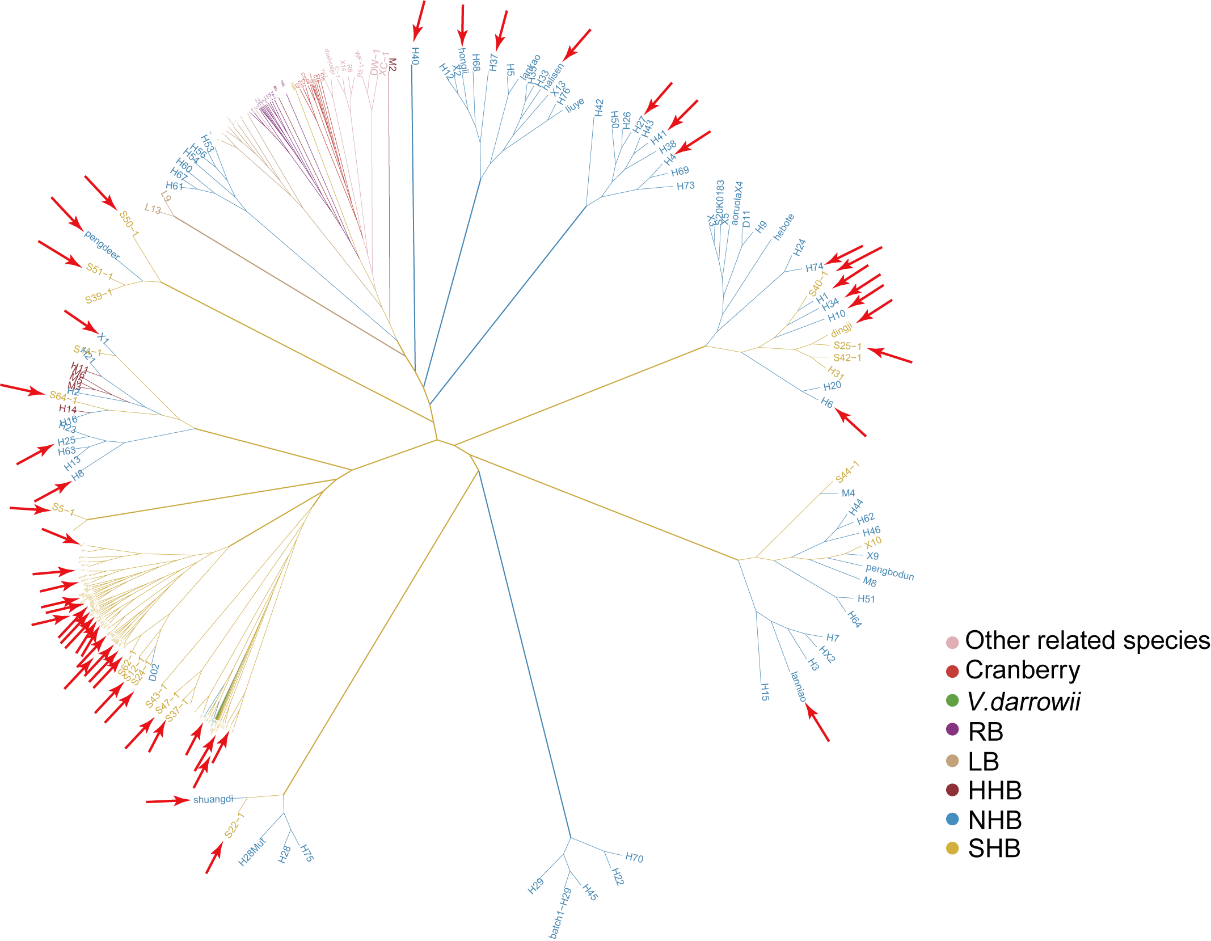
**

**Figure S7** The maximum likelihood phylogenetic tree of 222 blueberry accessions, with the selected 50 samples marked with red arrows.


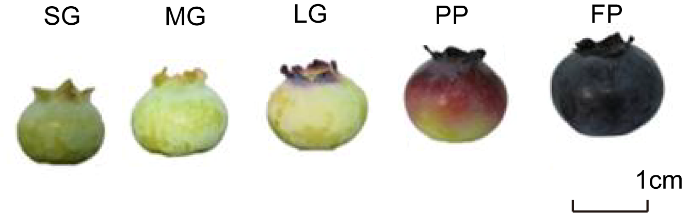


**Figure S8** Picture of blueberry fruits at five stages: small green (SG, 15 days after flowering), middle green (MG, 30 days after flowering), large green (LG, 45 days after flowering), partial purple (PP, 60 days after flowering) and full purple (FP, 75 days after flowering).


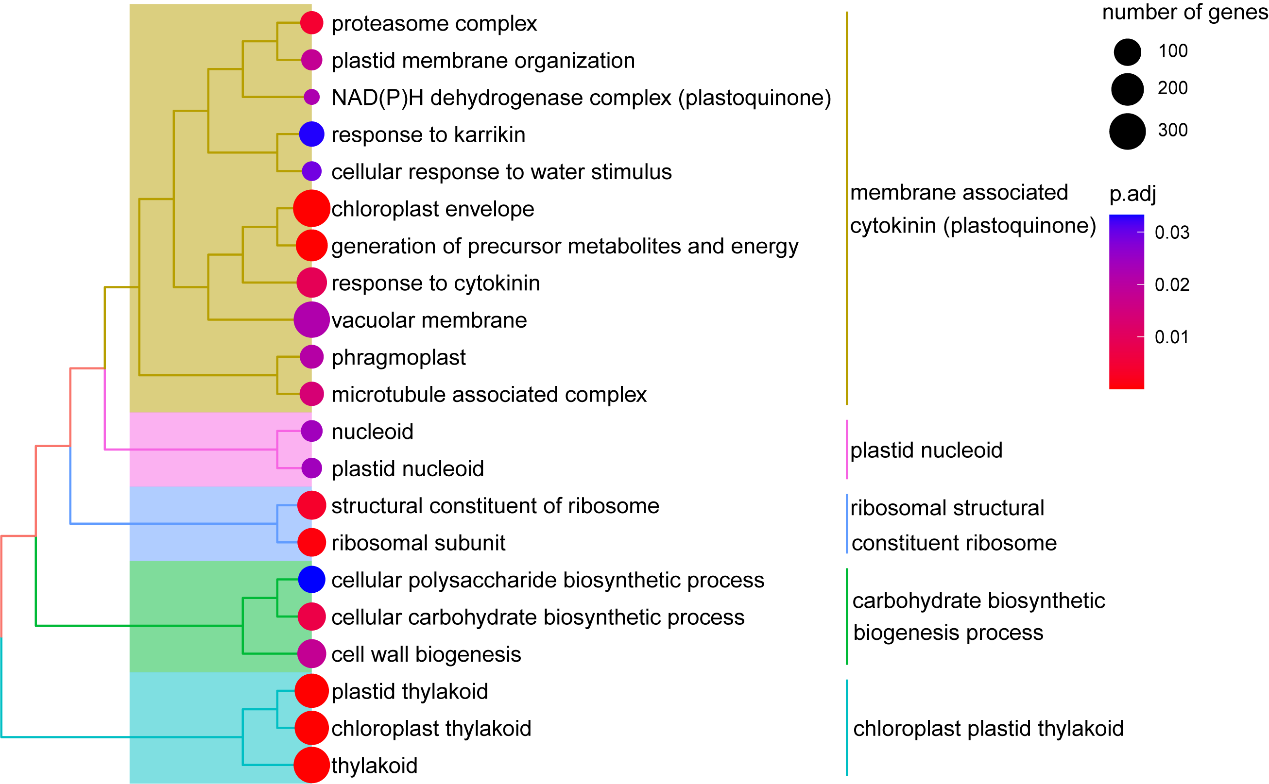


**Figure S9** Gene ontology (GO) enrichment of the differentially expressed genes during blueberry fruit ripening. The size of each dot represents the number of corresponding genes, while the color of the dot indicates the *p*-value associated with each GO term. GO terms are clustered using similarity indices.

**
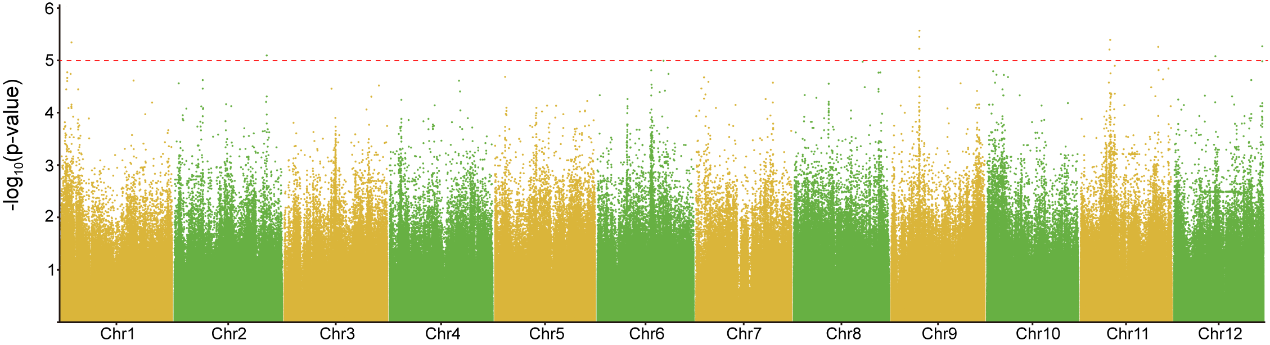
**

**Figure S10** Manhattan plot of the GWAS analysis of fruit firmness. The red dashed line represents the significance threshold (1e-5) for GWAS signals.


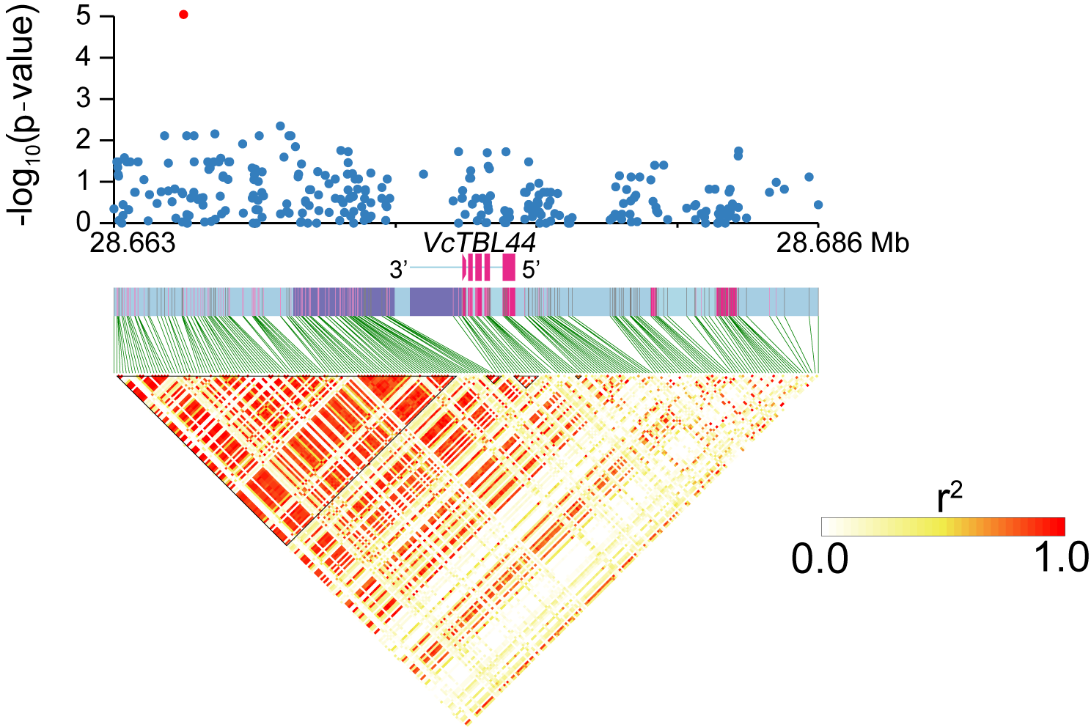


**Figure S11** Local Manhattan plot (top) and LD heatmap (bottom) surrounding *VcTBL44* on chromosome 8. The red dot represents the identified SNP significantly associated with fruit firmness.


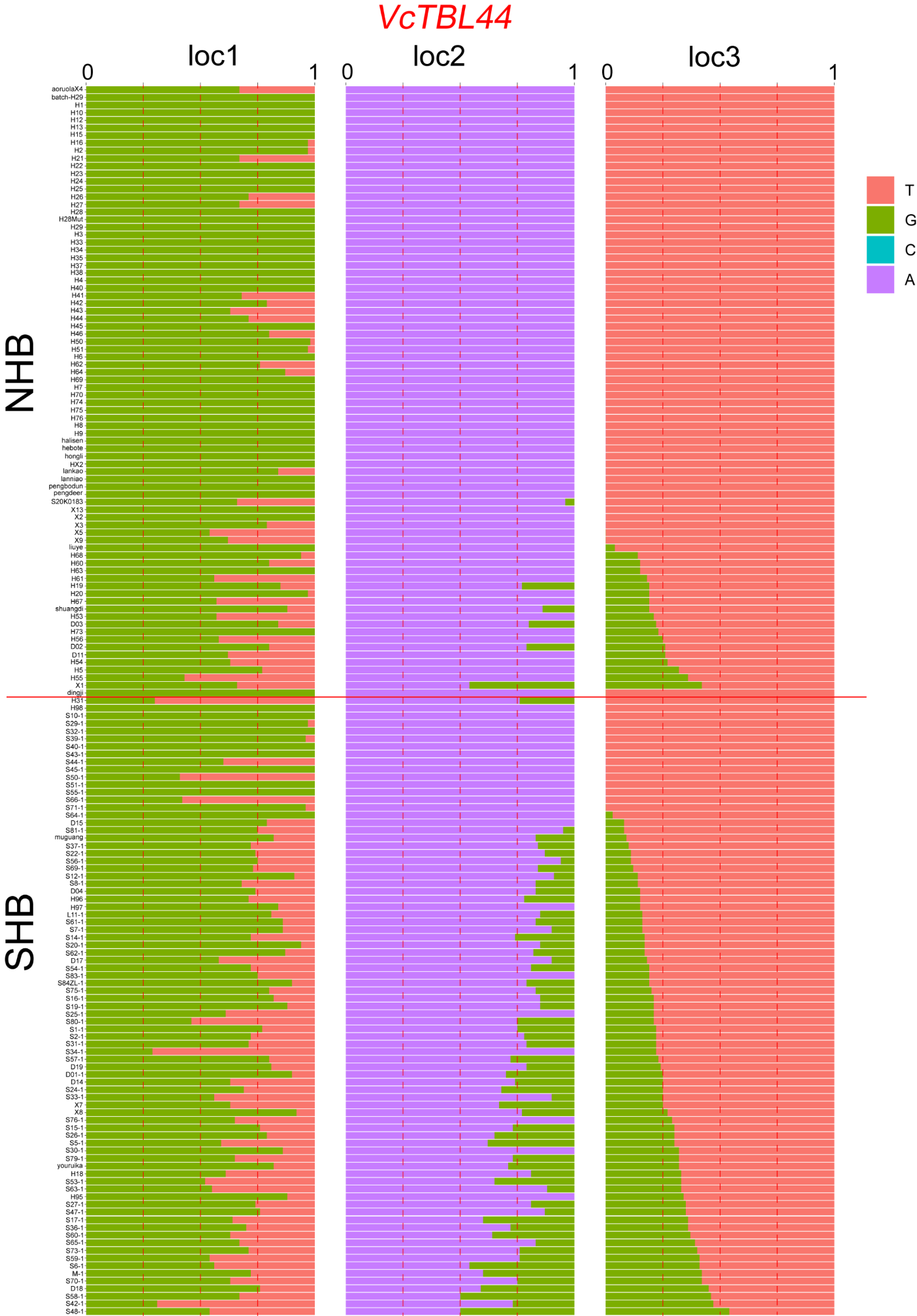


**Figure S12** The distribution frequency of three missense variations on *VcTBL44* in NHB and SHB subgroups. The location number is the same as in Figure 3D.


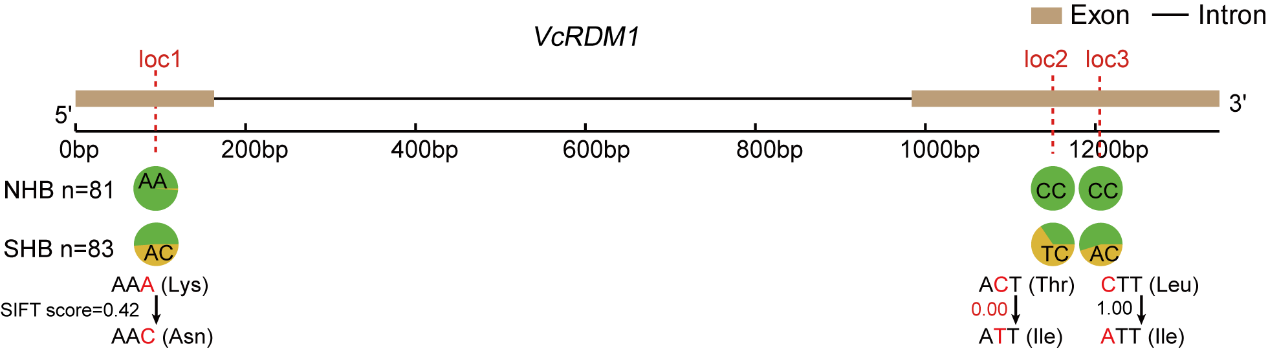


**1** Missense variants of *VcRDM1* and their allelic frequency in two blueberry subgroups. The positions of missense variants are indicated by the red dashed lines. The pie chart at each position represents allelic frequency within the NHB (upper) and SHB (lower) subgroups. Below each pie chart, variants at the codon level are displayed, and the specific nucleotide change is highlighted in red. The SIFT score was utilized to assess the impact of the variant on protein function, with smaller values indicating a greater impact. The value < 0.05 is considered to indicate a significant effect.

**
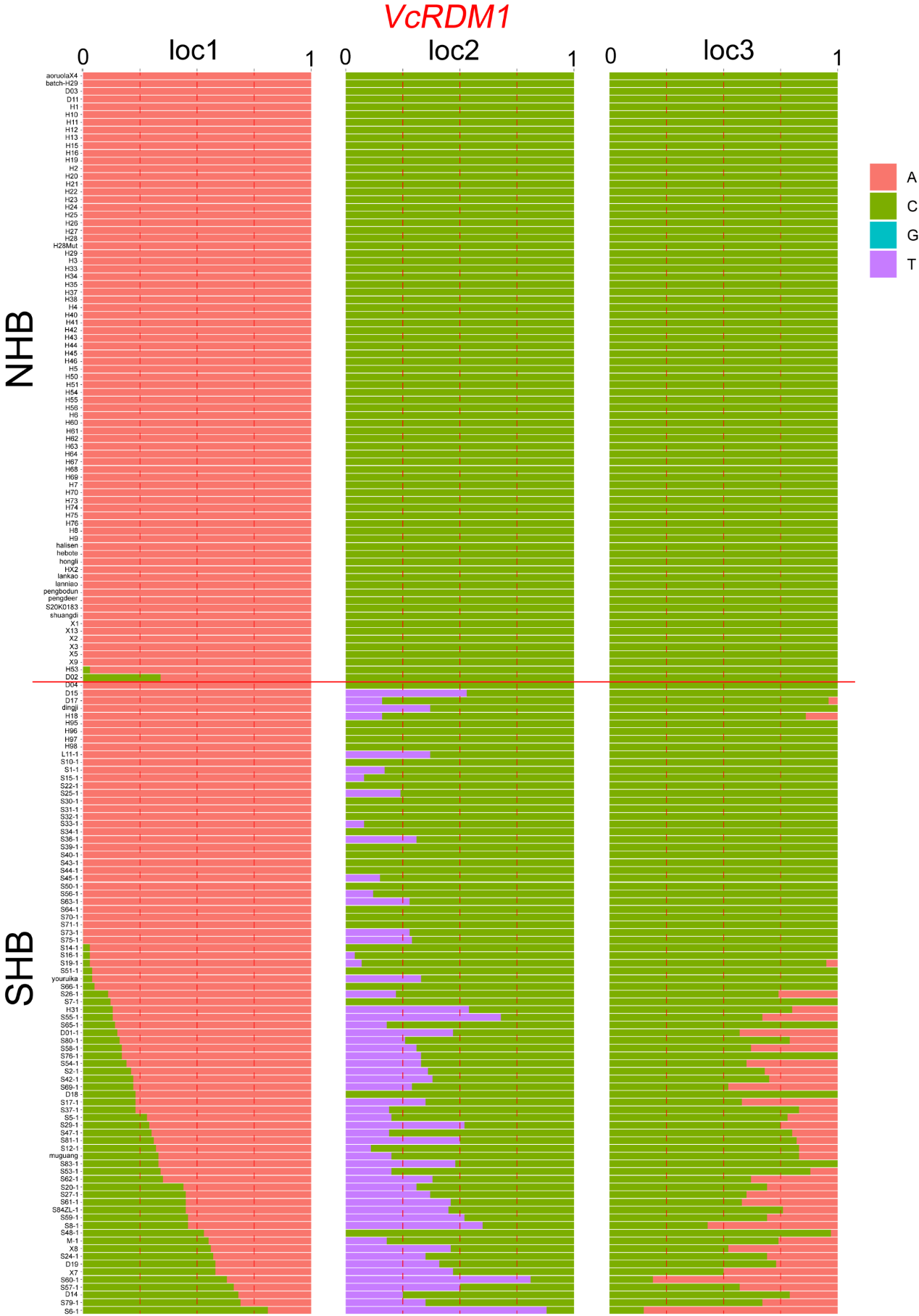
**

**Figure S14** The distribution frequency of three missense variations on *VcRDM1* in NHB and SHB subgroups. The location number is the same as in Figure S13.


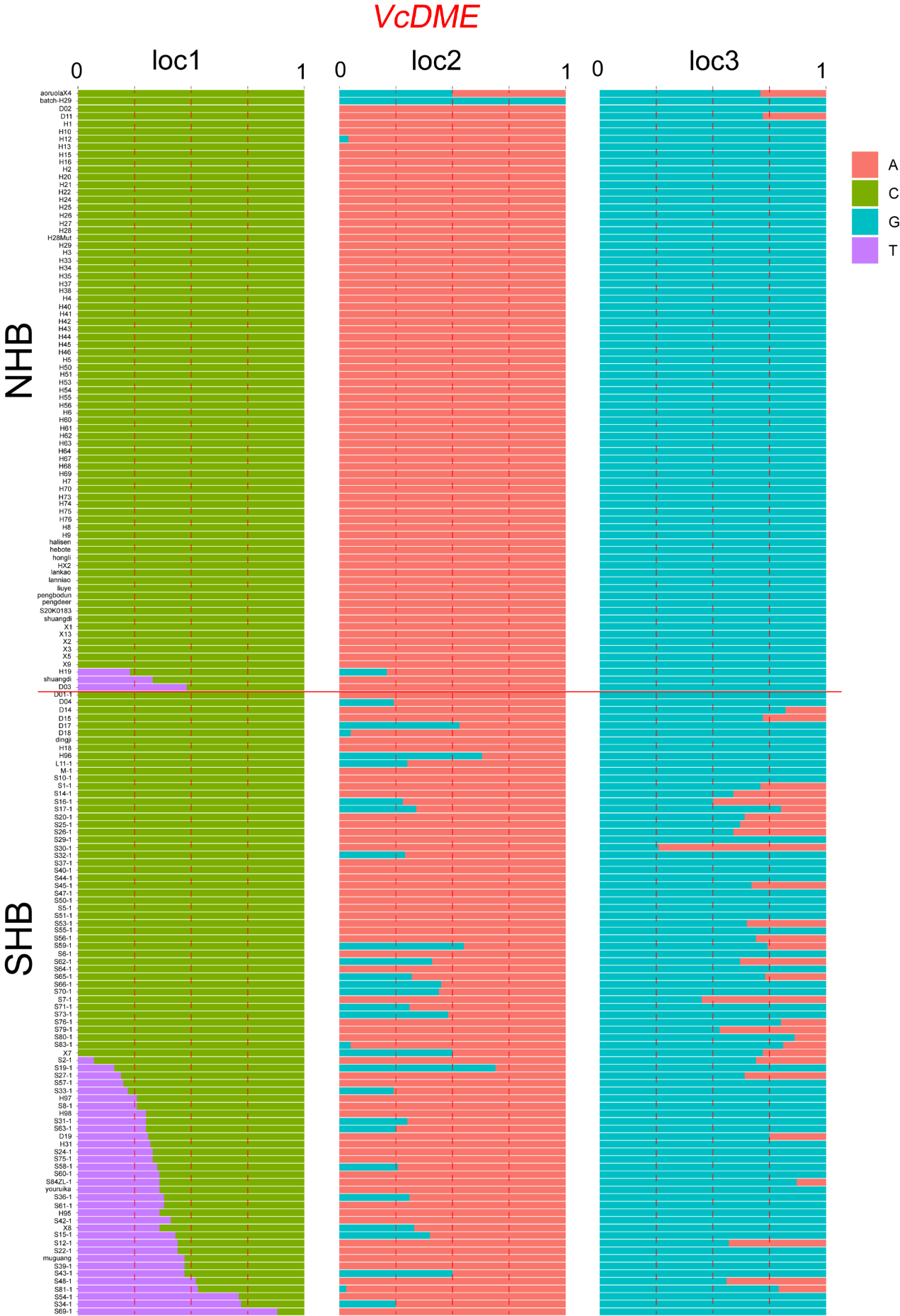


**Figure S15** The distribution frequency of three missense variations on *VcDME* in NHB and SHB subgroups. The location number is the same as in Figure 4A.


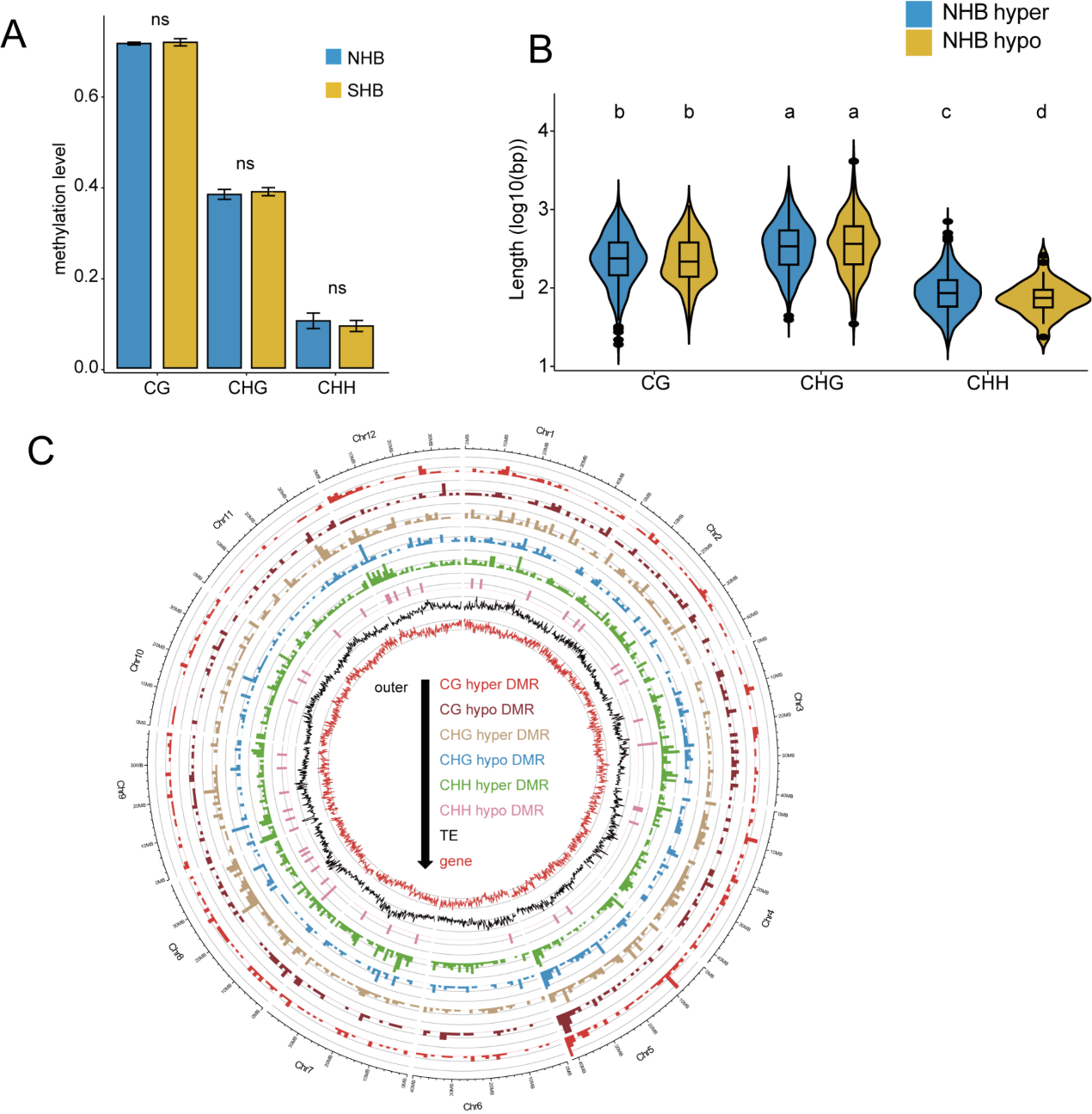


**Figure S16 Comparison of genome-wide DNA methylation between NHB and SHB. (A)** Global average DNA methylation levels of CG, CHG, and CHH in the NHB and SHB subgroups. Error bars represent the SD of the biological replicates. Statistically significant differences between the groups were assessed using Student's t-test. "ns" is used to indicate no significant difference. **(B)** Violin plots showing the distribution of lengths of different types of DMRs. Different letters above the boxes indicate significant differences (*p*-value < 0.05, Bonferroni correction) in multiple comparison testing. **(C)** Plots showing densities of CG hyper-DMRs, CG hypo-DMRs, CHG hyper-DMRs, CHG hypo-DMRs, CHH hyper-DMRs, CHH hypo-DMRs, transposable elements (TE), and genes are arranged from outside to inside. The outer rim displays the chromosome numbers and coordinates in megabases (Mb).


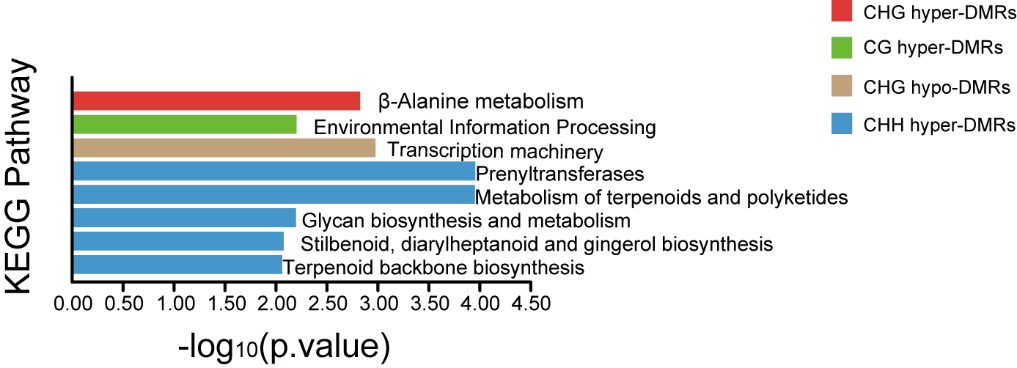


**Figure S17** KEGG analysis of genes found in differentially methylated regions (DMRs) between the NHB and SHB subgroups.


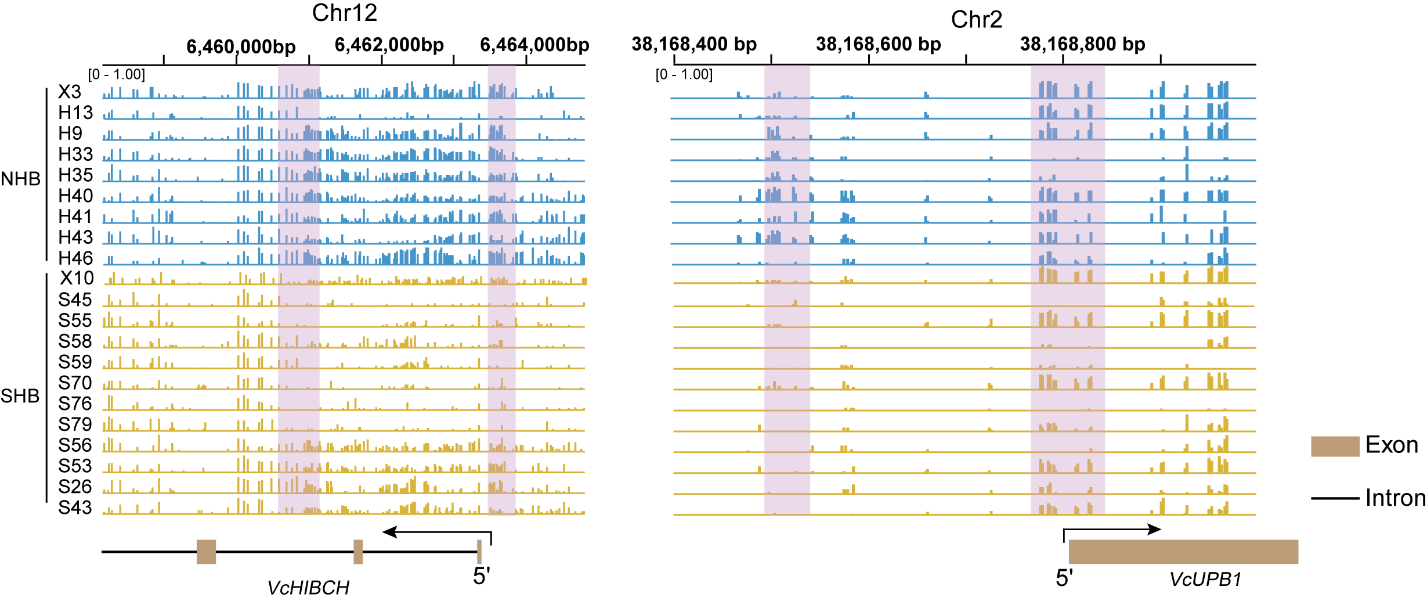


**Figure S18** Genome browser view showing the levels of CHG methylation of *VcHIBCH* (left) and *VcUPB1* (right) in nine NHB cultivars (upper, blue) and 12 SHB cultivars (lower, orange). Pink shading represents DMRs.


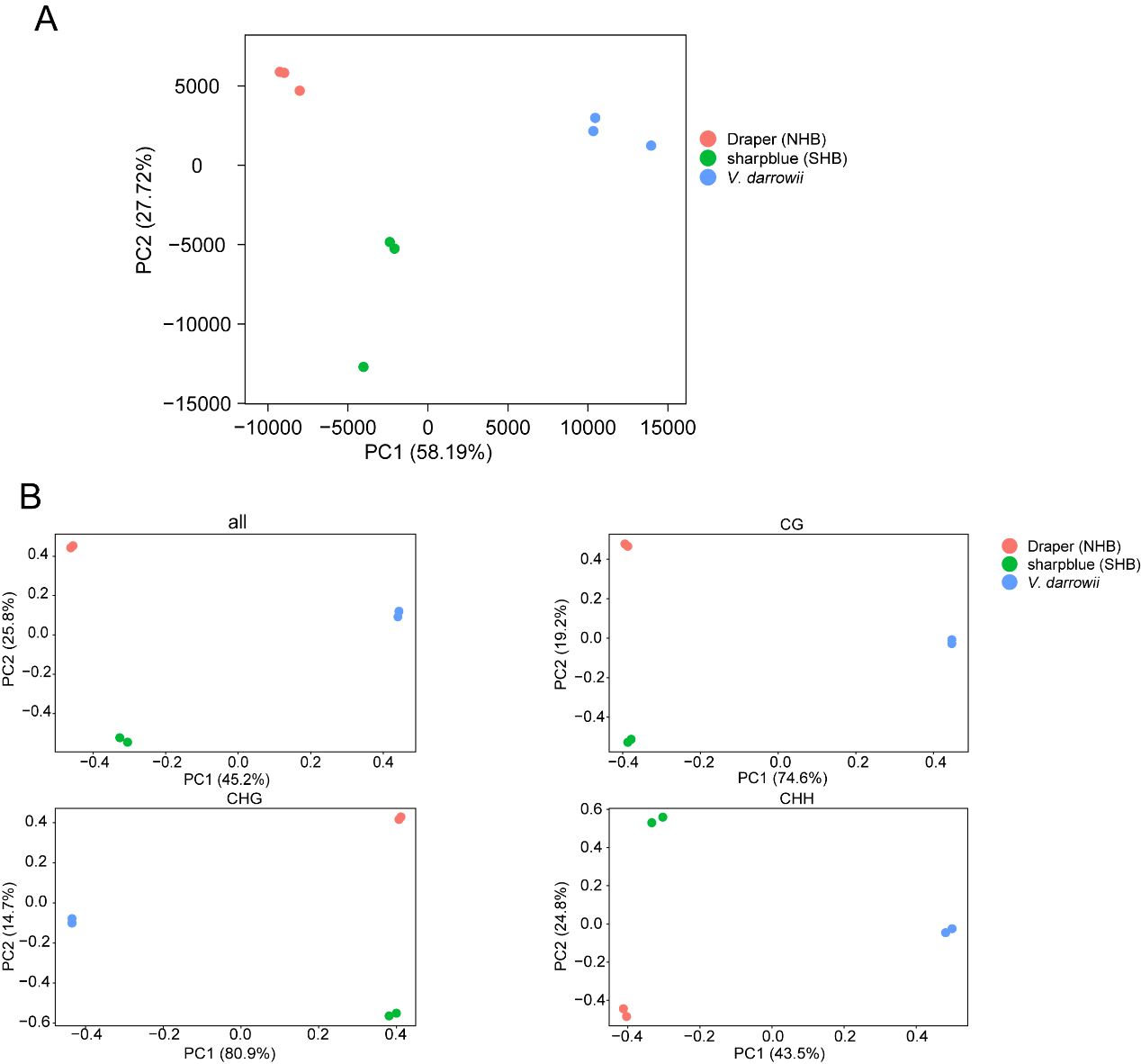


**Figure S19** Principle component analysis showing correlation between biological replicates of **(A)** RNA-seq and **(B)** whole-genome bisulfite-sequencing samples.


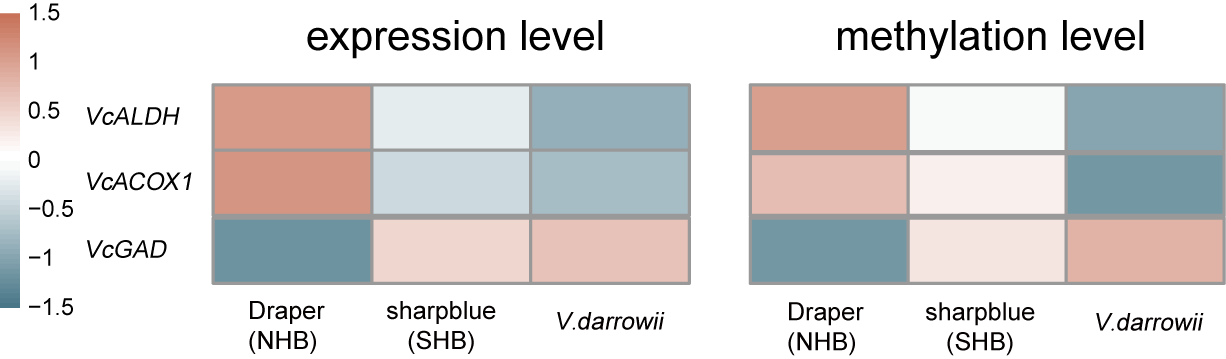


**Figure S20** Heatmap showing the relative expression levels (Transcripts Per kilobase of exon model per Million mapped reads, TPM) and methylation levels (upstream 1kb) of genes in the core β-alanine metabolism pathway.


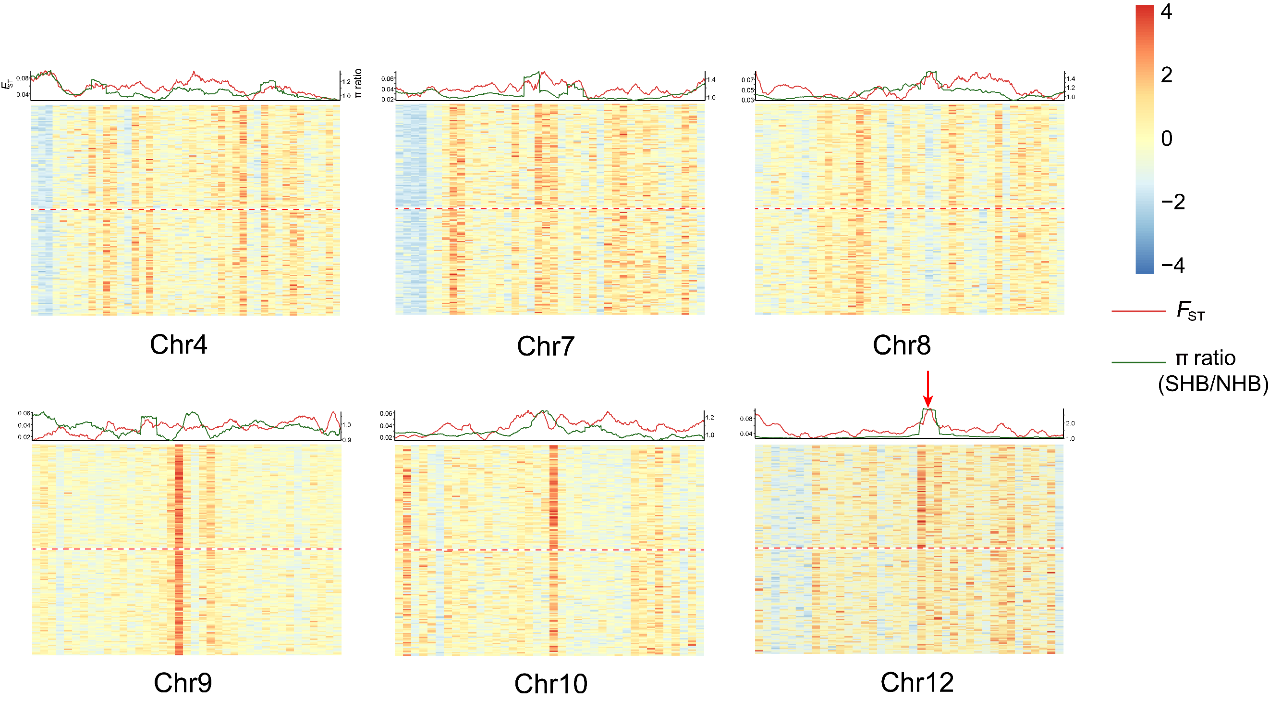


**Figure S21** Distribution of the density of TE insertions across chromosomes 4, 7, 8, 9, 10, 12, with the distribution of *F*_ST_ values (red curve) and π ratios (πSHB/πNHB, green curve) displayed above the heatmap.


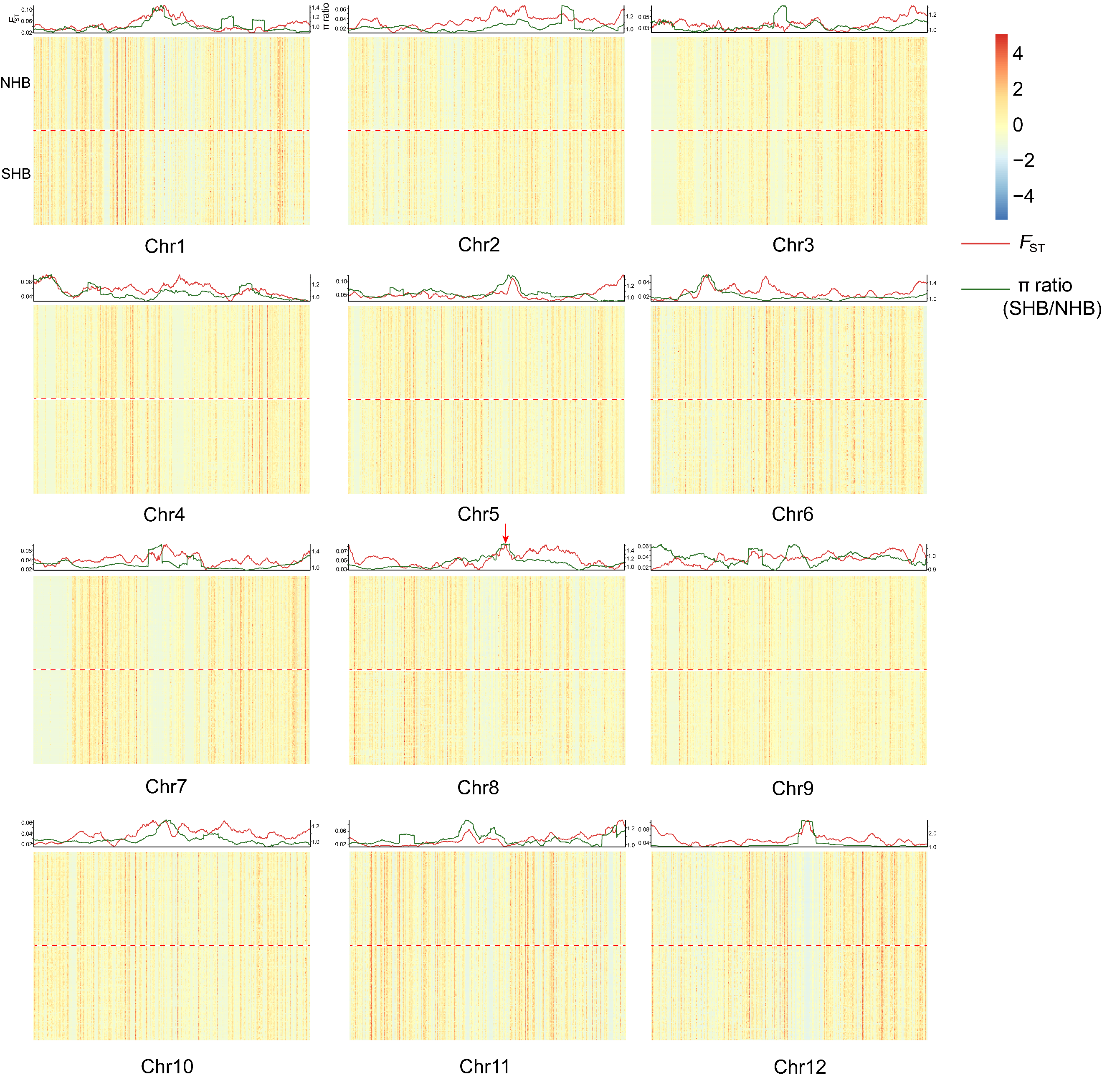


**Figure S22** Same as in Figure S21, except for TE deletion variations.


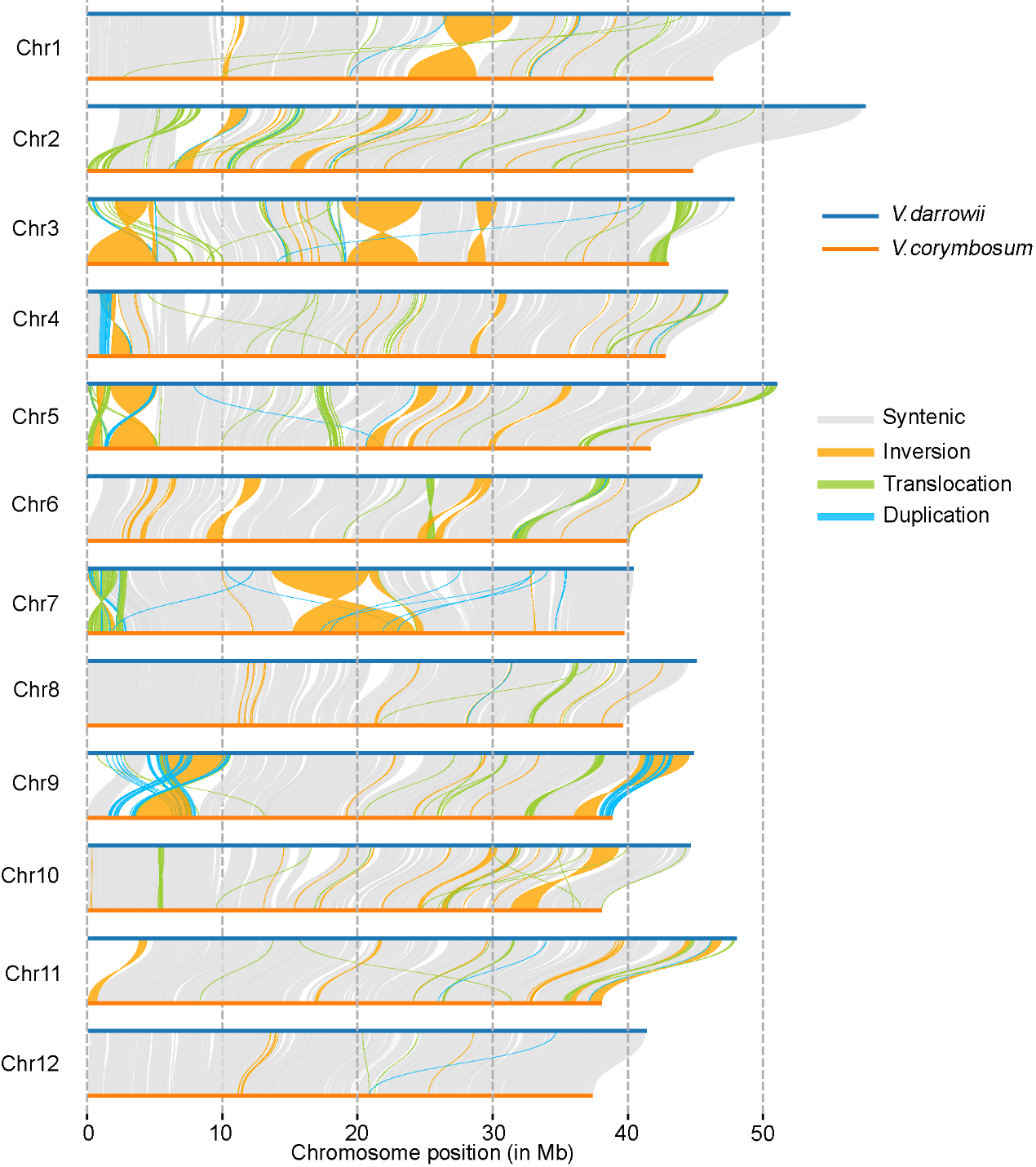


**Figure S23** Syntenic analyses between the assemblies of *V. corymbosum* ‘Draper’ (using the longest scaffolds representing each of the 12 homoeologous groups) and *V. darrowii.* The gray lines represent syntenic blocks, while the orange, green, and blue lines correspond to inversions, translocations, and duplications, respectively.


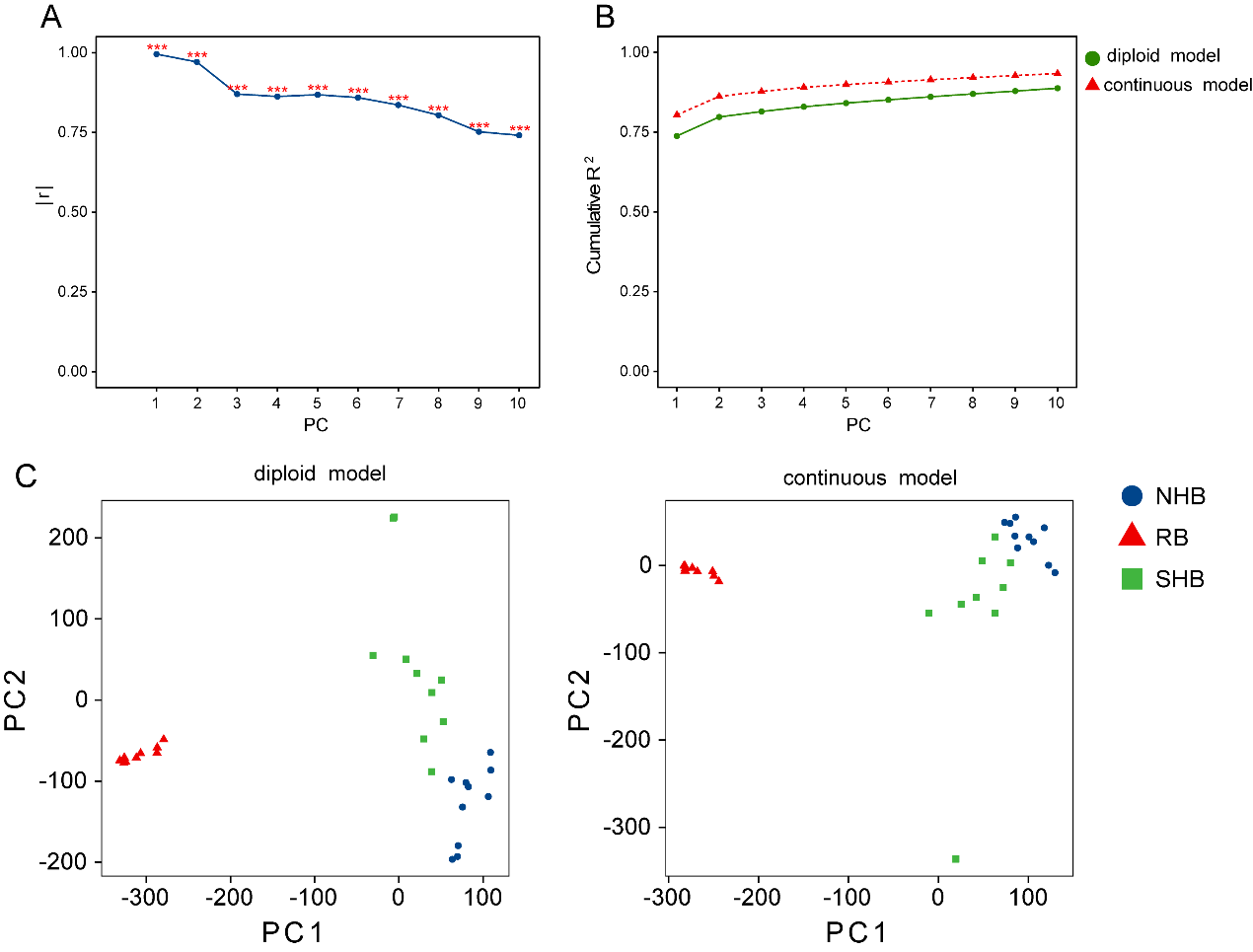


**Figure S24 Comparison of the diploid and continuous models. (A)** Plot of the absolute Pearson correlation coefficients for the principal component scores obtained from the continuous and diploid models. |r| represents the absolute value of the Pearson correlation coefficient. **(B)** The cumulative R^2^ of the top ten principal components calculated from the diploid and the continuous models. **(C)** Principal component analysis of 30 test accessions in diploid model and continuous model. PC1, first principal component; PC2, second principal component.


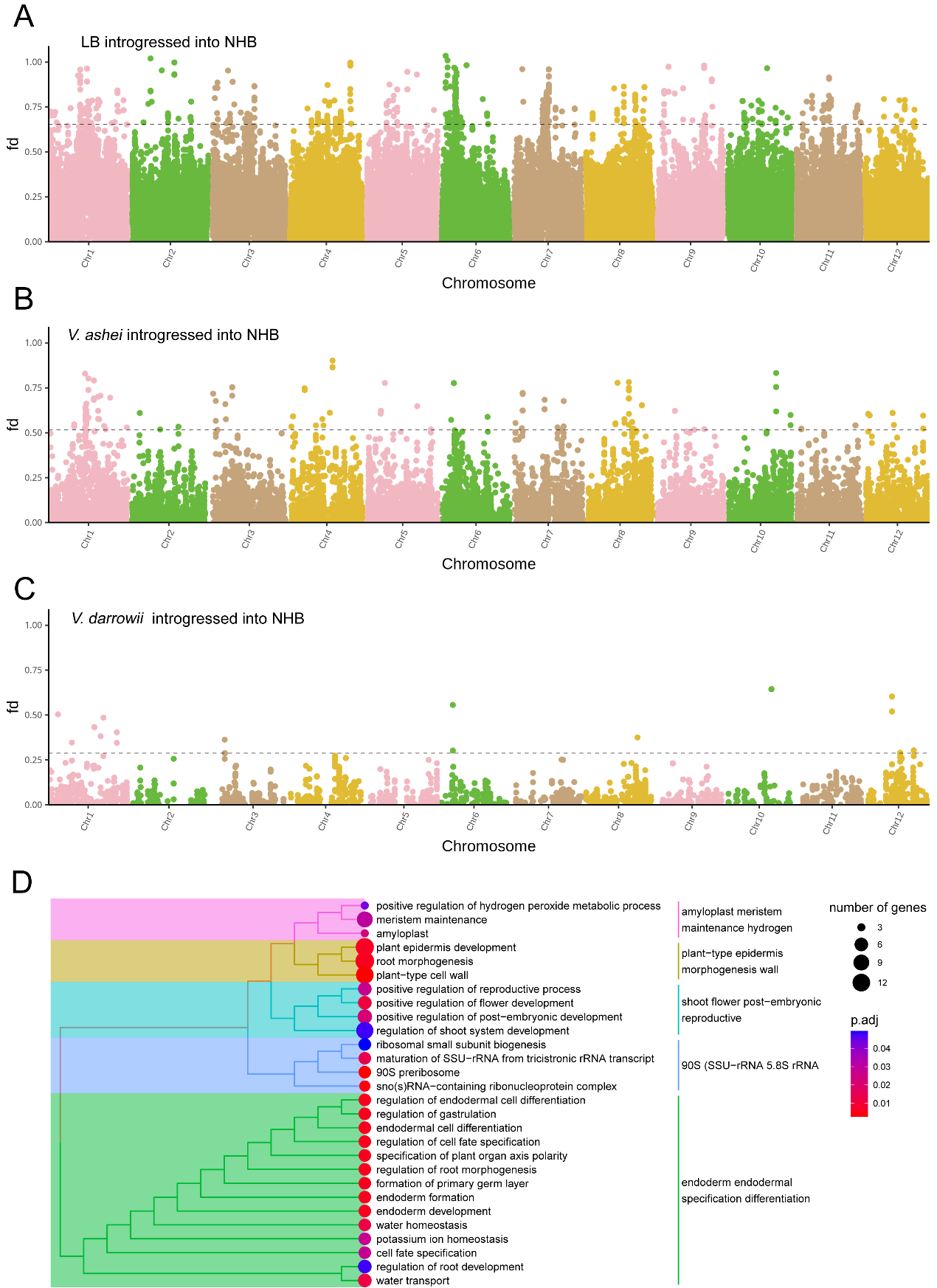


**Figure S25** **The gene introgression from other subgroups to NHB. (A)** Genome-wide distribution of f_d_ values (from LB into NHB) calculated for 50-kb sliding windows with a 5-kb step across the genomes. **(B)** Similar to Figure S25A, but depicting introgression from *V. ashei* into NHB. **(C)** Similar to Figure S25A, but depicting introgression from *V. darrowii* into NHB. **(D)** Gene ontology (GO) enrichment of the genes introgressed from LB into the NHB subgroup. The size of each dot represents the number of corresponding genes, while the color of the dot indicates the *p*-value associated with each GO term. GO terms are clustered using similarity indices.
